# Comparison of High Spatial Resolution PM_2.5_, PM_10_, and NO_2_ Estimates Using a Deep Ensemble Machine Learning Framework in a Low Pollution Setting

**DOI:** 10.1021/acs.est.6c02076

**Published:** 2026-06-30

**Authors:** Christine T. Cowie, Ivan C. Hanigan, Wenhua Yu, Cassandra Yuen, Karthik Gopi, Geoffrey G. Morgan, Nicolas Borchers-Arriagada, Jane Heyworth, Martin Cope, Lidia Morawska, Bin Jalaludin, Guy B. Marks, Yuming Guo, Luke D. Knibbs

**Affiliations:** † 104349 Woolcock Institute of Medical Research, Macquarie University, Macquarie Park, New South Wales 2113, Australia; ‡ South West Sydney Clinical Campus, UNSW Sydney, Liverpool, New South Wales 2170, Australia; § School of Population Health, 1649 Curtin University, Perth, Western Australia 6845, Australia; ∥ School of Public Health and Preventive Medicine, 2541 Monash University, Melbourne, Victoria 3004, Australia; ⊥ Sydney School of Public Health, and University Centre for Rural Health, 4334 The University of Sydney, Sydney, New South Wales 2050, Australia; # Menzies Institute for Medical Research, 3925 University of Tasmania, Hobart, Tasmania 7000, Australia; ¶ The School of Population and Global Health, The University of Western Australia, Crawley, Perth, Western Australia 6009, Australia; ∇ Commonwealth Scientific, Industrial & Research Organisation (CSIRO), Aspendale, Victoria 3195, Australia; ○ International Laboratory for Air Quality and Heath (ILAQH), School of Earth & Atmospheric Sciences, Queensland University of Technology, Brisbane, Queensland 4000, Australia; ⧫ School of Population Health, University of NSW, Sydney, New South Wales 2052, Australia; †† Burnet Institute, Melbourne, Victoria 3001, Australia; ‡‡ Public Health Research Analytics and Methods for Evidence, Sydney Local Health District, Camperdown, New South Wales 1450, Australia; §§ Centre for Safe Air, NHMRC Centre for Research Excellence, Hobart, Tasmania 7000, Australia; ∥∥ Global Centre for Clean Air Research (GCARE), University of Surrey, Guildford GU2 7XH, U.K.; ⊥⊥ Healthy Environments and Lives (HEAL) National Research Network, Canberra, Australian Capital Territory 2617, Australia

**Keywords:** machine learning, ensemble modeling, air pollution, exposure, particulate matter, nitrogen dioxide

## Abstract

Recent studies report improved performance of ensemble models over machine learning (ML) models for air pollution estimation, although there is little evidence of their added value in settings with sparsely monitored data. We developed and compared three ML models, a supervised linear regression (SLR) model, and an ensemble model, for estimating annual average PM_2.5_, PM_10_, and NO_2_ in NSW, Australia, a relatively sparsely monitored, low pollution, and large geographic region. We assembled pollutant data from government and project monitors and data on 236 predictors including land use, population, traffic, and satellite observations. We used a three-stage DEML framework: (1) base-ML models (Random Forest (RF), XGBoost, GBM) and a SLR model; (2) meta-learner (RF, XGBoost, GBM, GLMNet); and (3) ensemble. We reserved 10% of data for hold-out validation and conducted 10-fold Cross validation (CV) using the training data sets. We evaluated the models using CV-*R* ^2^ and RMSE. DEML models resulted in the best fit for all pollutants; however, improvements over base ML models were modest, indicating the latter, with lower cost of implementation, are valuable for low pollution settings with heterogeneous monitoring density. Choice of CV methods substantially impacted model performance and should be considered, along with setting constraints, when choosing modeling methods.

## Graphical abstract

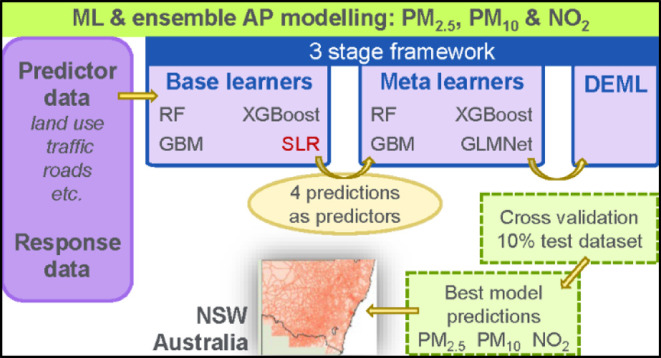

## 1 Introduction

Particulate matter (PM) and nitrogen dioxide (NO_2_) are two of the most extensively studied air pollutants with respect to human health, and are associated with a range of adverse outcomes including increased risk of cardiovascular and respiratory morbidity, metabolic disease such as diabetes, and premature mortality.^1–6^ PM toxicity is partially characterized by particle size, with finer particles less than 2.5 μm in diameter (PM_2.5_) considered to be more harmful than coarser particles (PM_10_).^7^

Epidemiological studies have relied on application of measured or modeled estimates of these pollutants to assign human exposures, with modeling techniques evolving rapidly over recent years from supervised linear regression (SLR) modeling (also known as land use regression (LUR))^8–10^ to increasingly sophisticated machine learning (ML) methods.^11,12^ The upsurge in the availability and quality of newly digitized and geocoded data has meant that vast data sets, such as satellite-derived air pollution measurements, and meteorological and land use data can be used as model inputs.^12–14^ The greater availability of predictor data for wider geographic areas has also meant that model outputs have been able to be applied over much larger spatial scales, such as whole continents or countries. This is advantageous if applied to nationwide or geographically dispersed cohorts,^15–17^ and even internationally to assess global impacts.^18–20^

ML methods have also been increasingly adopted due to expansion in the range and scope of predictor data and advances in data mining statistical techniques and computing resources.^12,21^ ML models are being progressively applied to describe air pollution surfaces as inputs for epidemiological studies. There has been particular attention to ensemble methods, which combine ML and other models in an effort to improve on singular models.^12,21^ Recently, ML and ensemble methods have been used to compare model fit in predicting PM mass and components and NO_2_ concentrations in the US,^22–24^ Europe,^25,26^ and in China.^15,17,27–29^

Advantages of using ML methods as predictive models include enabling use of all potential input data to help explain pollutant variability without prespecifying “explanatory” criteria which may limit “predictive” ability. ML models also better accommodate fitting of nonlinear relationships, are more resistant to multicollinearity and complex interactions, generally exhibit lower bias and reduce the high variance of predictions, offering an advantage over linear regression. They can also be used with relatively small training data sets. These are valuable attributes of an estimative model where the aim is to reduce prediction measurement error and hence minimize exposure misclassification in subsequent epidemiological analyses.

While there has been increasing application of ML methods,^11^ there are relatively few papers that have compared the performance and critiqued the practicalities of applying more sophisticated and complex methods using different modeling paradigms,^22,23,25–27,29,30^ and virtually none have done so in a low air pollution setting with a relatively sparse air pollution monitoring network as exists in Australia. This is an important research gap, given limited and competing resources in light of the availability of more complex and computationally demanding methods for pollutant prediction.

The above-mentioned modeling challenges of sparse monitoring outside of major metropolitan areas and a relatively low pollution setting where gradients in air pollution concentrations are small,^31^ informed the methods used in this study, where we have needed to estimate pollutant concentrations for an epidemiological study of long-term exposure on cardiovascular and respiratory outcomes in a >265,000 adult cohort living in NSW, Australia. In this study we have extended the previous methods used by us, including SLR, with^32,33^ and without^34–36^ satellite data, and a Bayesian maximum entropy model,^37^ to develop ML models using a deep ensemble ML (DEML) method,^38^ to estimate PM_2.5_, PM_10_, and NO_2_ in NSW, Australia. The issue of modest sample size (N) paired with a high number of predictors (P) is common in ML applications.^39^ Unlike statistical approaches focused on identifying explanatory variables, the prediction-focused algorithms of ML are robust even with modest sample numbers and can achieve high accuracy.^39^ We recently published results using this DEML method to predict ozone concentrations for the same study area of NSW, and reported that the model resulted in a small improvement in accuracy and precision compared to the base ML models tested.^40^

The aim of this paper is to compare model attainment of the DEML ensemble model with base ML models and an SLR model by determining estimative performance and error around the estimate of the cross-validated models. We also present model pollution estimates to describe population-wide exposures across NSW to PM_2.5_, PM_10_, and NO_2_. This is important as rural NSW is an area with sparse monitoring data, and the performance of ML modeling methods in such low pollution settings is not widely known.

## 2 Materials and Methods

### 2.1 Study Area

We collated air pollution data from a large area of southeastern Australia of 1,570,620 km^2^ (approximate land area denoted in Figure 1), consisting of: the whole of NSW; Canberra, the capital city of the Australian Capital Territory (ACT); a small area of southeastern Queensland, including the capital city of Brisbane; most of Victoria, including the capital city of Melbourne; and Adelaide, the capital city of South Australia (Figure 1). The data for states other than NSW were included in model development as they were thought to provide valuable additional information, as they were collected from monitored sites that were geographically close to regions in NSW where little monitoring takes place.

**Figure 1:**
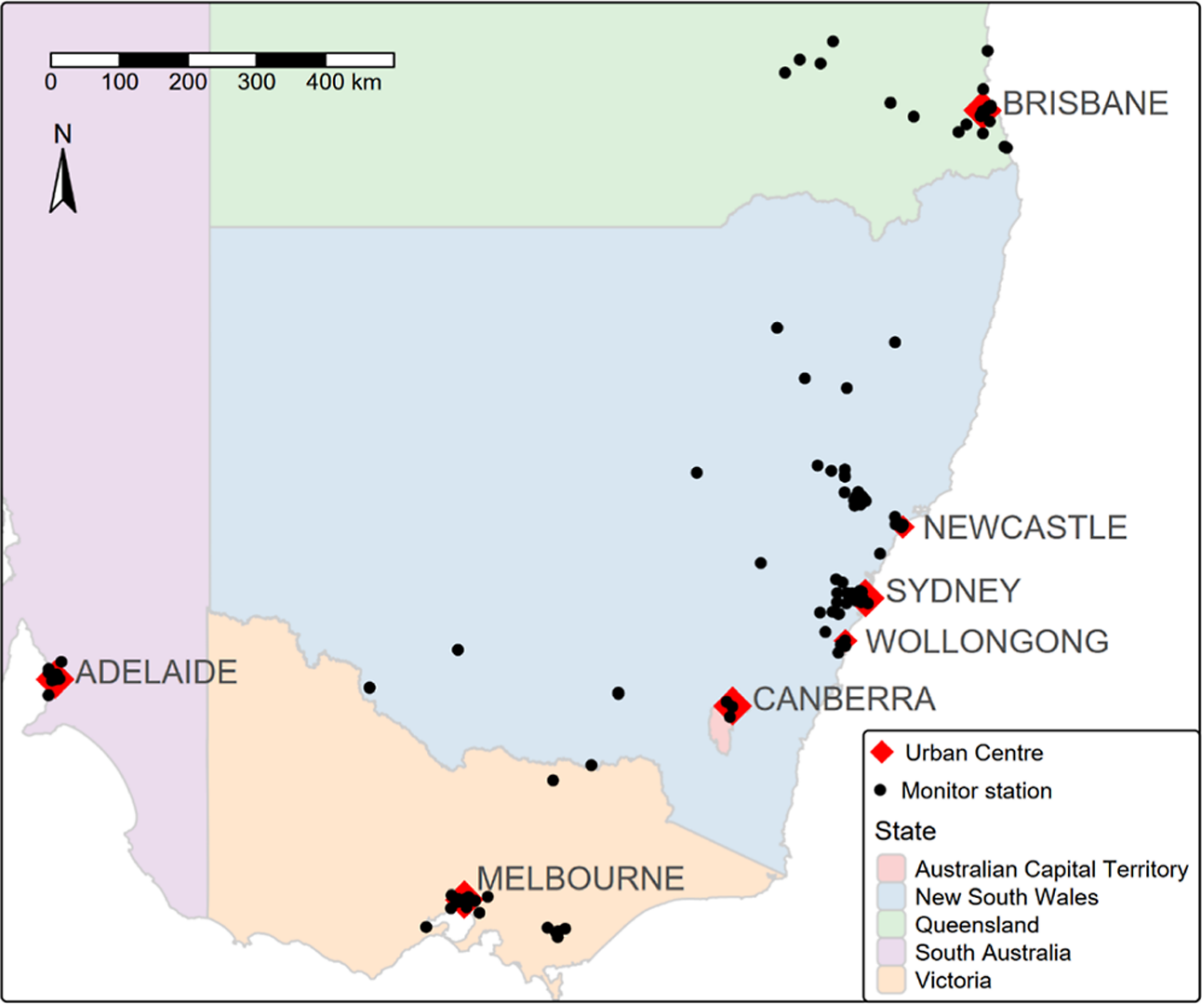
Map of government air pollution monitors used in our study for PM_2.5_, PM_10_, and NO_2_ (NSW, Victoria, ACT, South East QLD, and Adelaide, South Australia). Basemap.^47^

Once model development was finalized, we then predicted pollutant estimates for the whole of NSW, approximately 800,798 km^2^ in area,^41^ and climatically considered to be a warm, temperate zone. In 2024, NSW had a population of 8.5 million, comprising almost a third of Australia’s population.^41^ The Greater Sydney Area (GSA) includes the urban conglomeration of Sydney, the Central Coast, the Blue Mountains, and Wollondilly Shire areas, has a land area of 12,369 km^2^ and a population of 5.6 million in 2024.^41^ Outside the GSA and the urban centers of Newcastle-Lake Macquarie (407,357 population (2024)) and Wollongong-Illawarra (326,347 population (2024)), the remainder of the NSW population is concentrated in rural and regional towns or in sparsely populated areas to the west.

### 2.2 Air Pollution Data

We obtained data as the outputs (dependent variable) for air pollution modeling from two main sources: (1) government monitors which provided pollutant data with very good temporal resolution and (2) project monitored data collected from sites in the GSA and regional locations closer to Sydney to provide additional spatially resolved data for modeling, given that the larger proportion of the cohort resided in this area. The two sources of air pollution data are described as follows. We assigned latitude and longitude coordinates to each site using a geographic information system (GIS).

#### 2.2.1 Government-Monitored Data

Highly temporally resolved data were obtained from Department of Planning, Industry, and the Environment (DPIE) monitors from across NSW (Figure 1) for 2005 to 2018.^42,43^ Given our relatively sparse network of air quality monitors in rural and regional areas, we also obtained data for the three adjacent states of Queensland (to the north), Victoria (to the south), South Australia (to the west), and the ACT (Figure 1). This enabled our modeling domain to use observed data covering the whole state of NSW.

The number of government monitoring stations is given in Table 1. It should be noted that some of these monitors recorded measurements for different years within the study period due to varied decommissioning and commissioning of the monitors. Therefore, not all sites were monitored or had annual average data available for all of the years.

**Table 1:** Monitoring Site Types, Numbers, and Available Years of Data

pollutant	number of government monitors^a^ ^,^ ^b^	number of government monitors in Sydney metropolitan area^a^ ^,^ ^b^	number of field project sampler sites	number of valid annual average years per government monitor		
				min	max	median
PM_2.5_	76	15	41 (2018–2020)	1	14	4
PM_10_	93	18	41 (2018–2020)	1	14	7
NO_2_	71	18	39 (2006–2008)^c^	1	14	10
			47 (2013–2014)^c^			
			41 (2018–2020)^c^			

a Monitors whose data are used for compliance monitoring.

b Not all government monitored sites had valid (annual average) data for all study period years.

c Data from NO_2_ samplers deployed over three different projects during the study period (2006–2018).

PM_2.5_, PM_10_, and NO_2_ data from the regulatory monitors were provided at a 1 h resolution and nonconsecutive missing hourly data were imputed using the spline interpolation tool in the R package “imputeTS”.^44^ We then calculated annual means for years where there were more than 70% of hours observed in every calendar quarter.

The DPIE monitoring stations are accredited by the Australian National Association of Testing Authorities (NATA) and are operated in accordance with the Australian Standards (AS) method and the National Environmental Protection Measure for air quality regulation. The exception to this comprises some rural monitoring stations, where low-cost monitoring devices are used for indicative purposes. During the study period, the DPIE monitors for PM_2.5_ used Beta Attenuation Monitors (BAM) according to AS 3580.9.12, tapered element oscillating microbalance (TEOM) monitors, and gravimetric mass using BGI low volume samplers in accordance with AS 3580.9.10 (a reference USEPA method).^45,46^ NO_2_ is monitored in accordance with AS 3580.5.1 using a chemiluminescence analyzer and assuming NO_2_ to be the difference between measured total nitrogen oxides (NO_*x*_) and nitric oxide (NO).^45^

#### 2.2.2 Project Specific Measured PM and NO_2_ Data across the GSA and Nearby Regional Areas

The project monitors depicted in Figures 2 and 4 were deployed by the study team to capture additional spatial variation across the Sydney metropolitan area and nearby regional locations. The project monitors collected measurements for two-week periods in three different seasons from Nov 2018 to Mar 2020 (varied by site) using gravimetric methods for collection of PM and passive samplers for NO_2_, resulting in time-weighted average data for each two-week period. These data were then temporally adjusted to account for nonsimultaneous monitoring across sites and were then averaged to obtain annual average pollutant concentrations.

**Figure 2:**
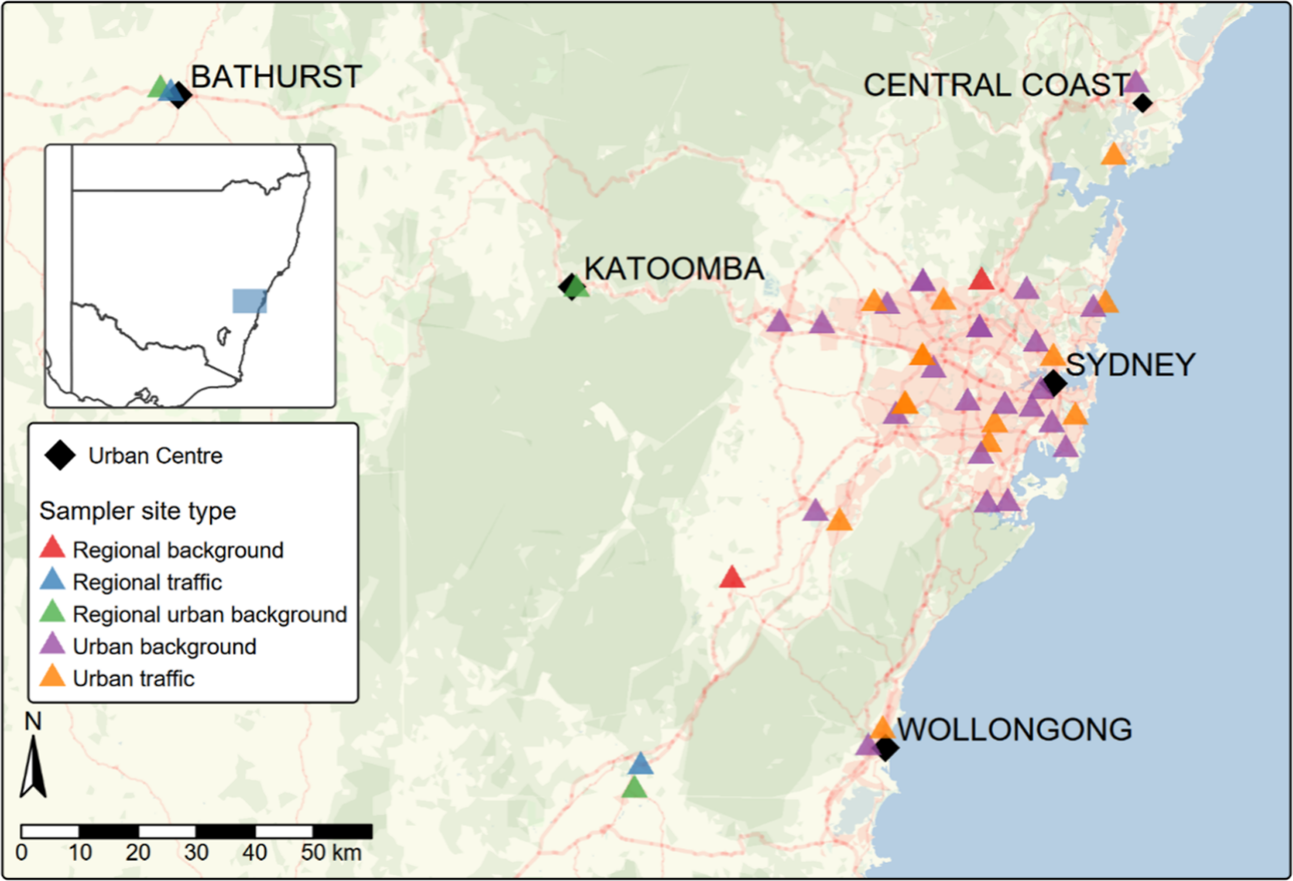
Harvard Impactor (HI) and Ogawa NO_2_ project sampler sites, GSA, Bathurst, Wollongong and Southern Highlands, with inset of the study area.

Samples were collected using HIs for PM (https://www.airdiagnostics.com/) and Ogawa passive samplers for NO_2_/NOx (https://ogawausa.com/passive-sampler/), at 42 sites in the GSA as well as Wollongong (to the south of Sydney), the Central Coast (to the north of Sydney), and in the regional towns/areas of the Blue Mountains and Bathurst (west of Sydney) and in the Southern Highlands (southwest of Sydney) (Figure 2), to obtain empirical measurements at a smaller spatial resolution than those afforded by the government regulatory monitors. We were unable to extend project monitoring beyond the locations specified above due to the limitation of resources needed to deploy across the vast land area, including remote regions, covered by NSW (800,798 km^2^). This project monitoring was conducted from November 2018 until March 2020, as outlined further below.

We followed the European ESCAPE protocol for measurement of the pollutants, choice of sampling sites, temporal adjustment, and averaging of concentrations to determine annual averages.^48,49^ We used HIs (MS&T, Air Diagnostics, and Engineering Inc., Harrison, ME) borrowed from the Institute for Risk Assessment Sciences (IRAS), Utrecht University, to collect PM on Teflon filters (Pall 37 mm, 2 μm pore size (Pall Corporation; R2PJ037)), according to existing ESCAPE study protocols for collection of PM^50,51^ and weighing of filters (IRAS, nd). Flow rates for the HI pumps were set at 10 L per minute and sampling was timed for 15 min per hour over each 14 day sampling period, to avoid filters becoming overloaded with matter. Flow volumes, adjusted for atmospheric pressure, were calculated and samples with <67% of sampling time were excluded.^50^ The filters were pre- and post-weighed in a humidity- and temperature-controlled laboratory using a microbalance with precision of 1 μg. Field blanks were deployed at the central Woolcock Institute of Medical Research (WIMR) site to determine the field limit of detection (LOD) and the average field blank concentration across the entire study period. The final PM concentration was calculated after subtracting the filter preweight, and adjusting for the control filters, and the average field blank measurements (IRAS, nd). The Supporting Information file includes a listing of the ESCAPE protocols followed in this study.

For NO_2_ and NOx, we used Ogawa passive samplers deployed at a height of 1.5 m next to the HIs and followed the ESCAPE protocol for deployment and measurement.^52^ Blank samplers were deployed every 10 samples to characterize the blank concentration for each batch, and the average of these blank sampler results was subtracted per batch of deployed samplers. Additional field blank samplers were loaded into zip-lock plastic bags and the brown Ogawa containers and taken into the field and exposed at the central WIMR site for each monitoring period, by opening the plastic bags but not removing the NO_2_/NOx badges. As for PM, field blanks were used to calculate the field LOD as three times the standard deviation (SD) of the field blanks. Preparation and analysis of the samplers were conducted at the University of New South Wales Mark Wainwright Analytical Centre laboratory by spectrophotometric determination of nitrate using the Saltzman method, with a calibration series of nitrite solutions prepared to obtain a calibration equation for each analysis run. Check samples were run every 10 samples as QA checks.

We chose sites that were representative of a range of expected population exposures based on local and prior knowledge of pollution sources gained from previous sampling campaigns. However, we did not sample in remote rural areas due to logistical constraints of travel, time, and cost, and typically, the populations in these areas are very small. Accordingly, we chose sites representative of urban background conditions (*n* = 21), urban traffic conditions (roads with >10,000 vehicles per day, vpd) (*n* = 12 except for one site with 9600 vpd), regional urban areas (*n* = 3 regional background; *n* = 2 regional traffic), and two sites (*n* = 2) representative of urban–rural interface exposures (Figure 2). One site was located at the WIMR, in Glebe, Sydney (rooftop of a five-story building), as the reference site, from which data were collected for all 2 week monitoring periods, and another site was colocated at a government regulatory monitoring site.

The HI and Ogawa samplers were colocated at each site for 2 weeks during each of three seasons: summer (Nov 2018–Feb 2019); autumn (Mar–May 2019); and winter (June–Aug 2019), with the equipment being rotated between sites.^48,49,51^ Given malfunction of the HI pump unit occurring on nine occasions (six sites during summer; three sites during autumn), we resampled at those sites for an additional 2 weeks during February 2020 (summer) and March 2020 (autumn) as needed. This 2020 timing was necessary to avoid sampling during the massive bushfire episodes experienced in the region during November 2019–January 2020.^53^ The monitoring program, therefore, provided three seasonal measurements for each site, which were then temporally adjusted using nearby government monitor data, to account for pollutant fluctuations driven by changes in weather due to nonsimultaneous monitoring across the sites.

For temporal adjustment of the project monitored data, the project sampling domain was divided into five subregions reflecting geography (i.e., Sydney central (GSA (excluding Coastal sites)) with longitude ≥150.94E); Sydney West (GSA with longitude <150.94E); Coastal (sites within 35 km to open ocean, including Wollongong and the Central Coast); West (Bathurst and the Blue Mountains; and the Southern Highlands), and a government reference station, operated by the NSW Department of Planning, Industry & Environment (DPIE) was assigned to each subregion. Data for each two-week period for both PM and NO_2_ were temporally adjusted using data from the assigned DPIE monitor from the relevant subregion and then averaged to provide an annual average concentration for each site. Methods for adjustment for both PM and NO_2_ were as previously described for NO_2_.^32^ These adjusted data were used as pollutant outcome data in all models in addition to the DPIE monitored data described in Section 2.1 above.

In addition to the above monitored data we also used NO_2_ data collected during previous passive sampling monitoring campaigns conducted in 2006–2008 ^54^ and in 2013–2014,^55^ to model NO_2_ in this study.

### 2.3 Predictor Variables

We chose potential predictor variables for modeling based on our previous work and a literature review. We then identified available data sets from both local (e.g., the Australian Government Public Sector Mapping Agency) and international sources (e.g., NASA) and obtained data in formats that could be implemented in a GIS. All GIS data were extracted for the monitor locations (project specific and government monitors) on a gridded overlay to enable pollutant estimation at an approximate 100 m resolution.

We obtained the following predictor data for model development: population and land use data from the Australian Bureau of Statistics (ABS) using mesh blocks, which are the smallest geographic unit available (approximately 30–60 dwellings containing 60–120 people on average); building heights and areas (Geoscape Australia); water cover from the MODIS satellite, NASA; traffic volumes from Zenith Pty Ltd. 2018 (count data) and PSMA (road networks); wood heater data (ABS); industrial emissions from the National Pollutant Inventory (NPI) (Department of Environment and Energy 2021); elevation from the Commonwealth Digital Elevation Model SRTM-derived; and weather and climate data from the Australian Bureau of Meteorology. Thirteen buffers ranging from 50 m to 10,000 m were used to create the land use, land/vegetation/water cover, population, emission sources, and some of the traffic-related variables. Satellite estimates of surface PM_2.5_ and NO_2_ were obtained from the Atmospheric Composition Analysis Group (2005–2018 data)^56^ and the OMI satellite (2005–2019)^57^ (see Supporting Information, Table S2, for details).

Predictor data were standardized by subtracting the mean and dividing by the SD using the data distribution of each predictor variable observed at the monitoring stations. We made an a priori decision to truncate values for the chosen traffic related predictor variables (e.g., distance to main road) to the range of data observed at the monitoring sites. These were truncated as per Wang et al., 2013, who showed that this procedure resulted in improved hold out evaluation *R* ^2^ for most of the pollutants studied.^58^ We also noted that it was routinely done in the ESCAPE studies to avoid unrealistic predictions arising from model extrapolations. For a full list of predictors and outcomes, and data sources used for the modeling please refer to Supporting Information Table S2.

### 2.4 Modeling Techniques and Model Building

We sought to obtain the best predictive models to estimate annual average pollutant concentrations for NSW for the period 2005 to 2018, using the government measured and project collected pollutant data (PM_2.5_, PM_10_, NO_2_) as the outcome variables. In summary, we used several statistical approaches, both in parallel and in series, to develop our final modeled PM_2.5_, PM_10_, and NO_2_ surface estimates. Each stage of the modeling procedure was designed to improve the accuracy of the preceding stage. For each pollutant, we used a separate DEML modeling framework to combine estimates from four base models and predictors, using the methods outlined in Yu et al., 2022.^38^ This comprised a three-level stacked ensemble model, which is considered an extension to the super learner framework.^59^ At each stage, measurements of PM_2.5_, PM_10_, and NO_2_ obtained from government monitoring stations (“monitor data”) and collected for our project (“project data”) were used as the dependent variables for selecting the best fitting model.

From the outset, both the outcome (measured air pollutant data sets) and the predictor variable data set were randomly split into a training data set (90% of data) and a test data set (10% of data). For the measured pollutant data (outcome variable), the random selection was across both temporal and spatial domains; i.e., data points were randomly chosen from all sites and all years. The test data set was held out from (not used for) model development and was only used for model evaluation at each stage (see 4.1 below). Figure 3 illustrates the steps used in the DEML modeling.

**Figure 3:**
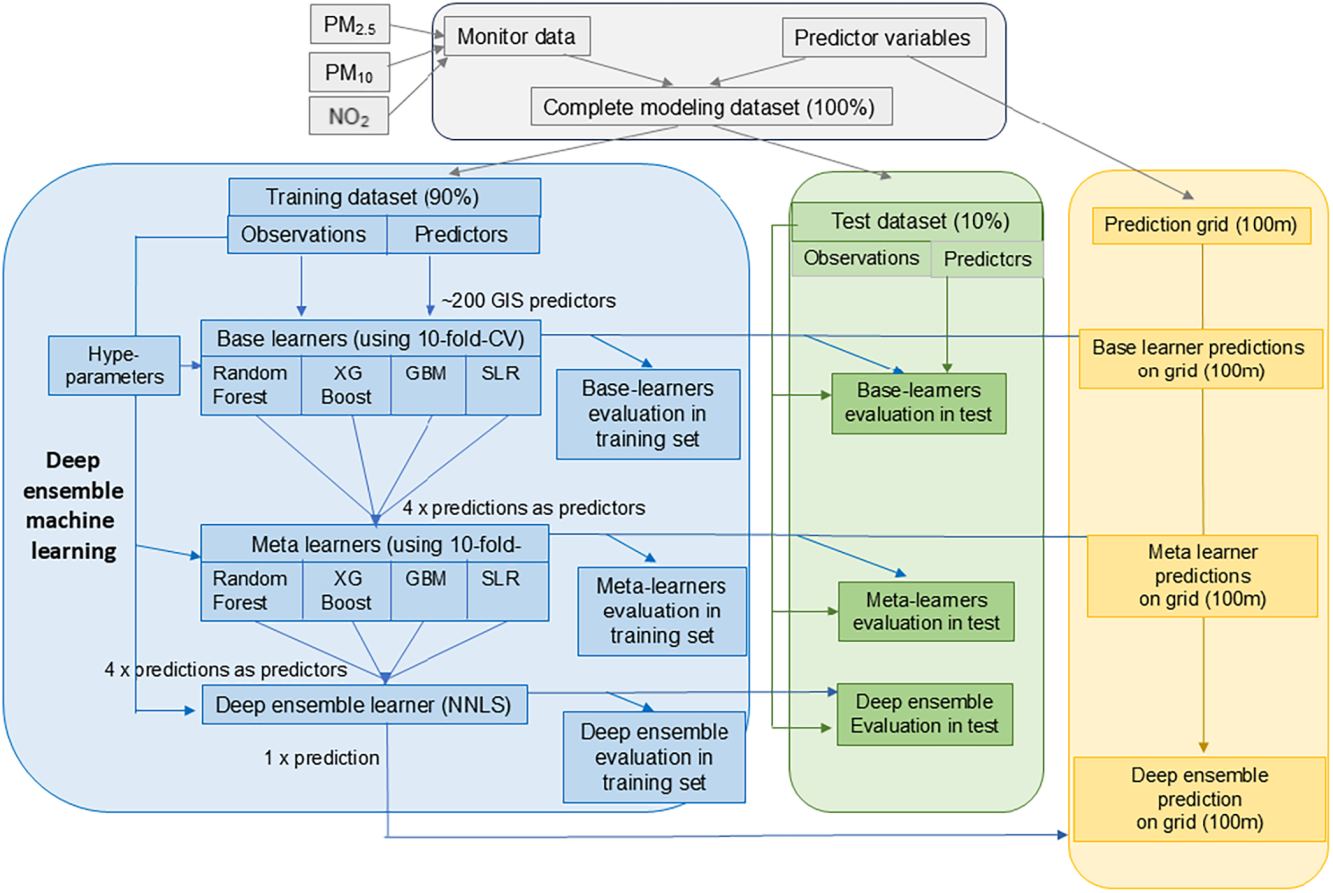
Deeper Ensemble ML (DEML) modeling framework used to estimate pollutant concentration.

Within the DEML process, we used three modeling stages including first, developing four base learner models comprising three ML models and a SLR method; second, we then combined these using meta-learner models, which optimally weight the predictions from each base learner; and third, we then used an ensemble model to combine the outputs of the meta-learner models.

In the first “base learner” stage, we used four models: an SLR model and three ML algorithms based on the nonparametric decision tree methodology:^60^ the Random Forest (RF) methodology,^61^ XGBoost,^62^ and GBM.^63^ Despite the growing use of ML models we decided a priori to include an SLR model as a base learner, as it remains a valid statistical approach to air pollution modeling, especially in more resource poor settings, and some studies have reported better model performance with the inclusion of SLR models compared to ML models.^64,65^ We used a forward selection approach for the SLR model. We used the implementations of the ML algorithms made available in the R “SuperLearner” package^59^ and initially identified optimal values of the hyperparameters to be used by the ML models by conducting a “grid search” systematically evaluating combinations of prespecified ranges of parameter values using 10-fold cross-validation to assess performance. The search evaluated multiple combinations of key parameters (e.g., tree counts, node sizes, learning rates) to minimize cross-validated prediction error. The final selected hyperparameter values are detailed in the Supporting Information file (Table S1).

Each of the base learner ML models were used to fit the predictors to the observed PM_2.5_, PM_10_, and NO_2_ data, and the DEML framework was applied to each pollutant separately. We excluded highly correlated candidate predictors by examining correlation matrices within variable groups (e.g., similar variables with different buffers) and then used a cutoff of 80% to identify variables for removal to avoid pairwise correlation, removing the variables with the largest mean absolute correlation. We also excluded variables that had the same value for more than 75% of observations. This resulted in 79 variables being used in the ML models. For the SLR models, standard supervised stepwise procedures, as implemented previously,^48,55^ were used and resulted in 13, 12, and 9 variables being retained by the PM_2.5_, PM_10_, and NO_2_ models, respectively (ESCAPE Study, 2010).

The outcome of the base learner stage was a set of four predictions (estimates) for each monitored site. The predictors were only used in the first “base learner” stage of the DEML procedure. We also calculated the relative influence (%) of the predictor variables for the best predictive models in the “base learner” stage and calculated the difference between the relative influence and the mean relative influence (diff_mean) as well as the difference between the relative influence and the median relative influence (diff_median). We produced plots of the relative influence of the predictor variables for each pollutant using a cutoff of diff_median >0. The calculation of relative influence differed by model type with the method outlined in Friedman (2001) applied to the GBM models^66^ and calculation of “gain” applied to the XGBoost model.^67^

In the second stage, the four prediction estimates (from the base learners) were used as predictors and fed into another set of model algorithms used as meta-learners. Each of the meta-learners was used to fit these new “predictors” to the observed PM_2.5_ and PM_10_ and NO_2_ monitored data (as the ground truth). Four separate methods were used as meta-learners: three nonparametric ML methods, RF, XGBoost and GBM; and one Generalized Linear Model fitted using penalized maximum likelihood using the GLMNET R package.^68^ This resulted in four predictions (estimates) for each air quality monitored site.

The final ensemble stage of the DEML approach was the application of the non-negative least-squares (NNLS) algorithm to combine the four estimates from the second meta-learner stage into a single prediction (estimate). The NNLS optimization algorithm obtained the set of coefficients that minimized the prediction error. The set of weights for meta-models in the final ensemble must meet predefined constraints, but there is no requirement for each of the meta-learners to be included. The details of these methods are described elsewhere.^69^

The final step was pollutant estimation at gridded location points using a resolution of 100m and using the best performing model.

R software was used for all of the analysis with the “deeper” R package used to run the DEML modeling.^38^

#### 2.4.1 Model Evaluation

At each stage of the modeling procedure, we evaluated the final models using 10-fold cross-validation within the training data set (that is, the 90% of data used to train the models) and also applied the fitted models to the 10% held-out test data set, withheld randomly across the spatial and temporal domain. This means that all sites including those in sparsely monitored areas were included in the cross validation (CV) process. Random hold-out validation was chosen to make the best use of our limited and sparsely located rural data, being a valid technique where data points are sparse, as it maximizes the data available for model training. We used random CV as our primary CV method of choice and undertook spatial CV as a sensitivity analysis. While we acknowledge that random CV is likely to yield overly optimistic predictive *R* ^2^ and RMSE, compared to spatial CV, some researchers have noted that spatial CV often produces overly pessimistic *R* ^2^ and may not be best suited where data are limited.^70,71^ For both the training and held-out test data sets, the accuracy of the models was estimated by calculating the *R* ^2^ and the root-mean-square error (RMSE), as was done in our recent modeling for ozone.^40^

## 3 Results

### 3.1 Monitored Pollutant Data and Predictor Variables

Monitors were distributed across the study region as shown by Figure 4. Monitor types and their temporal coverage are described in Table 1.

**Figure 4:**
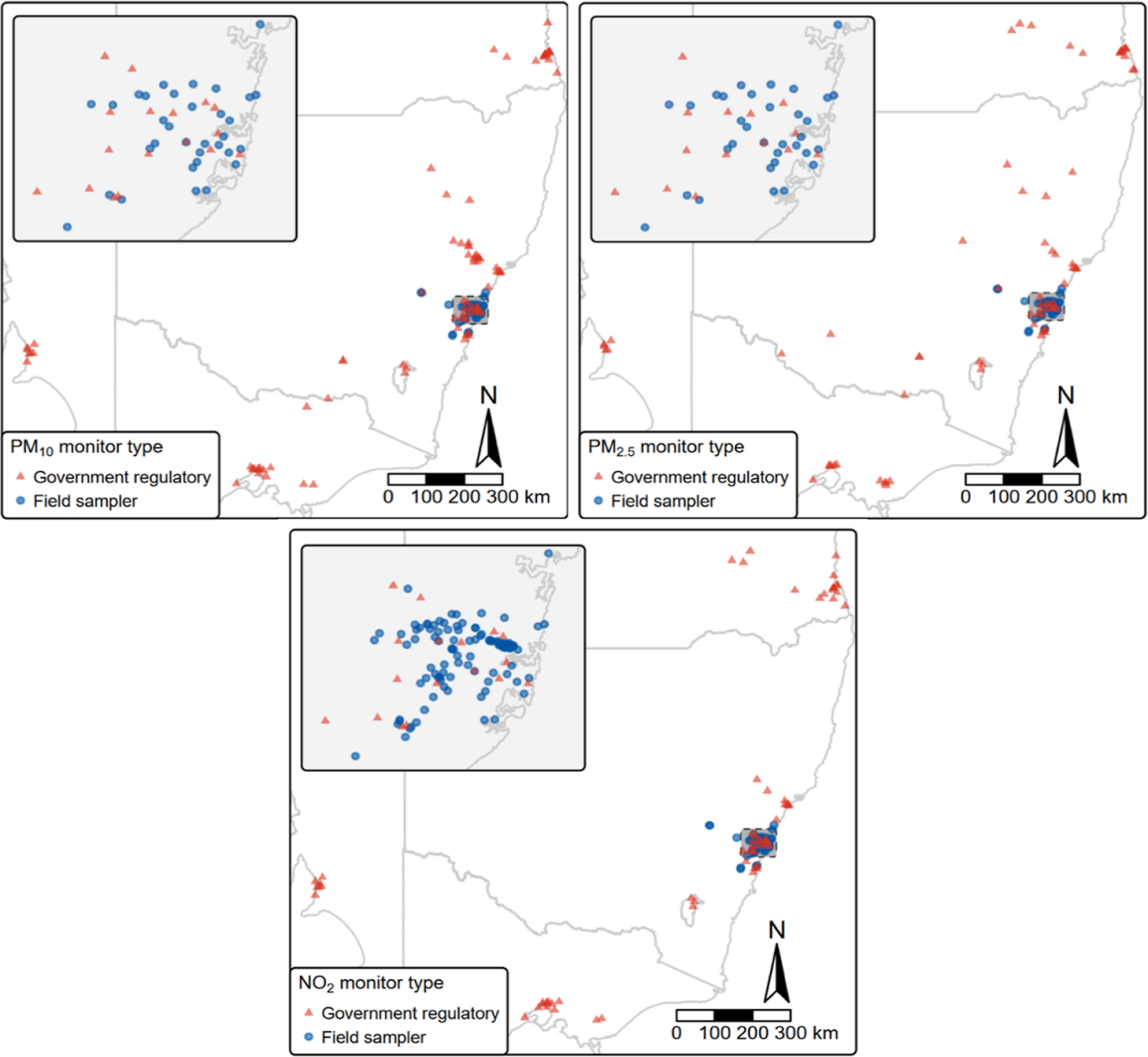
Distribution of government regulatory monitors (red triangles) and project samplers (blue circles) used for modeling across southeastern Australia for pollutants PM_2.5_, PM_10_, and NO_2_ with an inset map of the Sydney area.

The mean pollutant concentrations in the study area for the period 2005–2018 were: PM_2.5_ = 6.9 μg/m^3^ (range 5.7–7.5 μg/m^3^); PM_10_ = 17.8 μg/m^3^ (range 15.2–22.8 μg/m^3^), and for NO_2_ = 7.9 ppb (range 6.5–10.5 ppb). Descriptive statistics for each of the pollutants are provided in Tables S4–S6.

We identified data for 236 candidate predictor variables of which 79 were selected after filtering out highly correlated variables and those without substantial variation in observed values. See Tables S2 and S3 for details of data preparation for all predictors. These 79 variables were offered to both the ML and the SLR models for model development. Table 2 indicates the number of variables retained in each model: 79 for each of the base ML models and 9–13 variables for the SLR models, subject to the pollutant being modeled.

**Table 2:** Base Learner Evaluations for PM_2.5_, PM_10_, and NO_2_

(a) PM_2.5_					
		training (*N* = 372) 10-fold cross-validation		held-out testing (*N* = 40) sample^a^	
model	variables	*R* ^2^	RMSE (μg/m^3^)	*R* ^2^	RMSE (μg/m^3^)
GBM	79	0.57	0.94	0.44	1.21
XGBoost	79	0.61	0.90	0.60	1.03
Random Forest	79	0.60	0.91	0.46	1.20
SLR	13	0.50	0.98	0.41	1.26

(b) PM_10_					
		training (*N* = 702) 10-fold cross-validation		held-out test(*N* = 76) sample^a^	
model	variables	*R* ^2^	RMSE (μg/m^3^)	*R* ^2^	RMSE (μg/m^3^)
GBM	79	0.85	1.48	0.80	1.97
XGBoost	79	0.84	1.56	0.80	1.91
Random Forest	79	0.71	2.18	0.66	2.45
SLR	12	0.41	2.80	0.16	3.35

(c) NO_2_					
		training (*N* = 722) 10-fold cross-validation		held-out test (*N* = 80) sample^a^	
model	variables	*R* ^2^	RMSE (ppb)	*R* ^2^	RMSE (ppb)
GBM	79	0.92	0.96	0.90	1.17
XGBoost	79	0.91	1.02	0.90	1.20
Random Forest	79	0.88	1.16	0.84	1.55
SLR	9	0.81	1.44	0.82	1.60

a Training CV: the *R* ^2^ and RMSE are the averaged 10-fold cross-validated predicted values for the algorithms using the 90% training data set; the test sample represents the predicted values for the held out 10% sample.

### 3.2 Base Learner Models

The training data set (79 predictor variables) was used in the first step of the DEML modeling process. This entailed fitting and evaluating the four base-learning models. The values of the hyperparameters that were used in fitting the ML models are shown in Supporting Information Table S1.

In the training data set used to fit the PM_2.5_ model, the cross-validated (CV) *R* ^2^ and RMSE ranged from *R* ^2^ = 0.61 (RMSE = 0.90 μg/m^3^) for the best fitting model (XGBoost) to *R* ^2^ = 0.50 (RMSE = 0.98 μg/m^3^) (SLR model). *R* ^2^ values were similar for the held-out 10% test samples for the XGBoost model, but were lower for the other three models (Table 2a). The RMSE increased for all models when using the held-out 10% test data sets, although the RMSE for the XGBoost model was substantially lower than for the other models (Table 2a).

For PM_10_ and NO_2_ modeling, the GBM and XGBoost models performed the best and were similar with respect to *R* ^2^ and RMSE. For PM_10_, the CV GBM model performed the best, but the XGBoost model performed better in the held-out test sample with the same *R* ^2^ value of 0.80 but a lower RMSE (Table 2b). For the NO_2_ modeling, the GBM performed slightly better in both the CV data set (*R* ^2^ = 0.92 RMSE = 0.96 ppb) as well as the held-out test data set (Table 2c).

For all pollutants, the SLR model was the weakest performer with the lowest *R* ^2^ and highest RMSE for both the CV and test models. The SLR models performed poorly for PM_10_ but reasonably well for NO_2_ in the test data set.

Figure 5 shows the frequency distribution of pollutant predictions for each pollutant using the base learner models training data sets. It indicates that the prediction distributions were very similar for all models including the SLR model.

**Figure 5:**
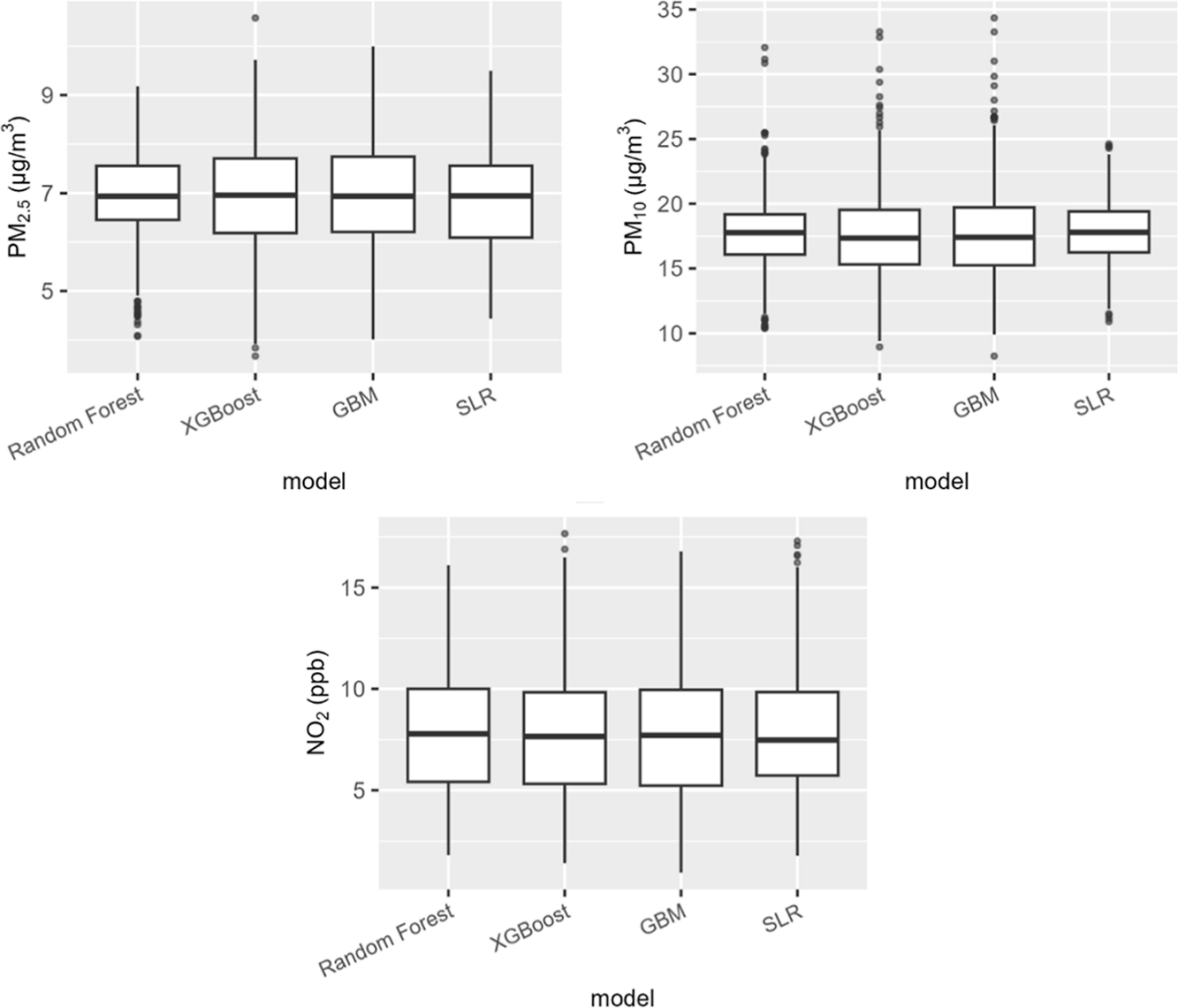
Boxplots of base learner predictions on the training set for PM_2.5_, PM_10_, and NO_2_.

Figures S1–S3 in the Supporting Information show the relative influence of predictors for the best performing base-learner ML models for each of the pollutants, that is, GBM models for PM_10_ and NO_2,_ and the XGBoost model for PM_2.5_.

### 3.3 Meta Learners and the DEML Model Evaluation

The evaluation data for the metalearners and the deep ensemble NNLS models are shown in Table 3. This table shows the weighting of the meta-learner estimates used in the DEML models and the evaluation statistics for the final deep ensemble NNLS models for both the CV training NNLS models and the held-out test NNLS models.

**Table 3:** Meta Learners and DEML Model Evaluation for PM_2.5_, PM_10_, and NO_2_

(a) PM_2.5_			training (*N* = 372) CV		test (*N* = 40) sample	
model type	model	weights for NNLS (ensemble)	*R* ^2^	RMSE (μg/m^3^)	*R* ^2^	RMSE (μg/m^3^)
second step Meta models	GLMNET	0.975	0.63	0.87	0.57	1.07
	Random Forest	0	0.59	0.91	0.53	1.12
	XGBoost	0.002	0.50	1.05	0.39	1.30
	GBM	0.023	0.61	0.89	0.51	1.13
DEML	NNLS	NA	0.64	0.85	0.56	1.07

(b) PM_10_			training (*N* = 702) CV		test (*N* = 76) sample	
model type	model	weights for NNLS (ensemble)	*R* ^2^	RMSE (μg/m^3^)	*R* ^2^	RMSE (μg/m^3^)
second step meta models	GLMNET	0.932	0.86	1.46	0.81	1.90
	Random Forest	0.069	0.84	1.54	0.82	1.86
	XGBoost	0	0.79	1.81	0.76	2.09
	GBM	0	0.83	1.61	0.77	2.05
DEML	NNLS	NA	0.87	1.39	0.81	1.90

(c) NO_2_			training (*N* = 722) CV		test (*N* = 80) sample	
model type	model	weights for NNLS (ensemble)	*R* ^2^	RMSE (ppb)	*R* ^2^	RMSE (ppb)
second step meta models	GLMNET	0.726	0.92	0.95	0.90	1.16
	Random Forest	0.274	0.91	0.98	0.89	1.25
	XGBoost	0	0.89	1.12	0.86	1.37
	GBM	0	0.91	0.98	0.88	1.28
DEML	NNLS	NA	0.94	0.80	0.90	1.18

Table S7 in the Supporting Information shows the results of the spatial CV for both the base learner ML models and the second stage meta-learner models. These results indicate that the spatial CV *R* ^2^ were lower and the RMSE were higher for all models, compared to the random CVs.

### 3.4 PM_2.5_, PM_10_, and NO_2_ Exposure Maps

Figure 6 illustrates annual average PM_2.5_, PM_10_ and NO_2_ concentrations predicted by the DEML model for NSW for 2015 (chosen as a representative year), including an expanded representation of the Sydney metropolitan area. The maps indicate that the highest estimated concentrations for all pollutants occur in the Sydney area. The highest PM_2.5_ exposures appear to occur along the road network and predicted concentrations across NSW range from 1.1 to 11.8 μg/m^3^. Predicted concentrations ranged from 8.6 to 30.3 μg/m^3^ for PM_10_ and from −2.1 to 24.6 ppb for NO_2_. These ranges in modeled estimates are comparable to monitoring data from the regulatory monitoring stations and project monitors (refer to Tables S4–S6), although the range for the modeled estimates is, as expected, slightly larger.

**Figure 6:**
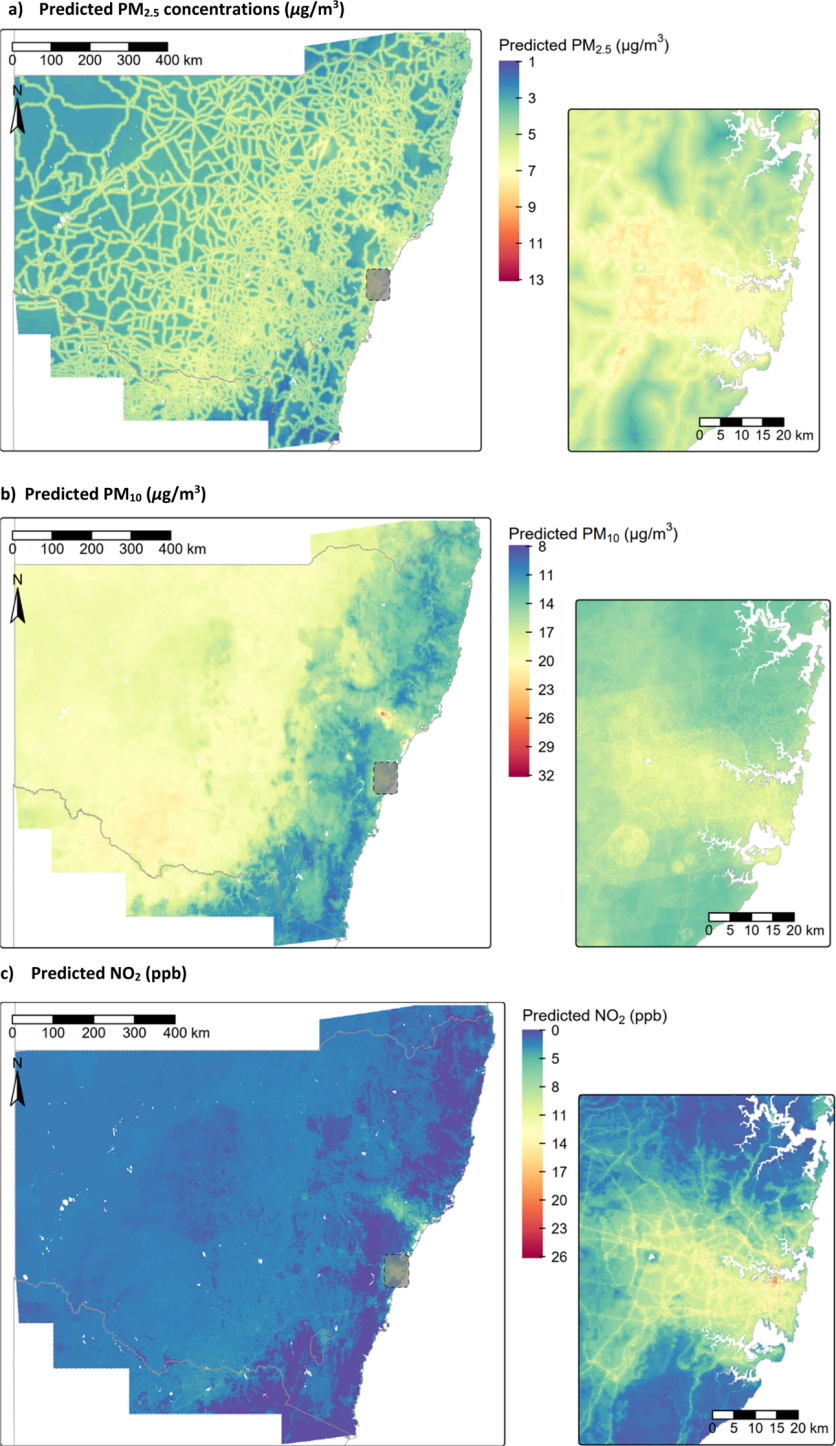
DEML model predicted annual average (a) PM_2.5_, (b) PM_10_, and (c) NO_2_ concentrations for NSW and ACT, with a focus on the Sydney metropolitan area (inset maps), for 2015.

It is important to note that while negative pollutant concentrations for NO_2_ (as estimated in the DEML model) are physically impossible, it is possible for models to make negative predictions due to extrapolation of predictive variable values beyond the range measured, for example, at semiremote and remote locations. We believe that for NO_2_ estimation, the predictor variable “tree cover” chosen by the SLR model was likely to be responsible for the DEML estimated negative NO_2_ concentrations, as the values for this variable were not originally truncated to the range of observed values, as was done for the traffic related variables (see Section 2.3 above). Tree cover has not previously been found by us to impact on NO_2_ estimation in this way and so we had no a-priori reason to truncate its values prior to model building. We therefore made the posthoc decision to reset all negative NO_2_ estimates to a threshold limit of zero to correct for model extrapolation beyond the training data range and to ensure all predictions remained physically plausible. Given that virtually none of the health cohort to which these estimates will be applied live in the areas with extremely low NO_2_, we believe that the impact in subsequent epidemiological analyses will be very small.

## 4 Discussion

In this study, we found that the final DEML model resulted in the best model fit for all pollutants (PM_2.5_, PM_10_, and NO_2_) when compared with individual models at preceding stages of the framework, however, the DEML results were only marginally improved over the best ML base learner models for PM_10_ (GBM) and NO_2_ (GBM) and were similar to the best meta-learner (2nd stage) model (GLMNET).

This is interesting and perhaps unexpected, given our air pollution monitoring network is relatively sparsely distributed over a large rural geographic area. This contrasts with other regions like Europe, China, and North America where this type of work has previously taken place, but which have the advantage of much denser monitoring networks. To highlight the differences in monitor numbers, our models used data from 117 PM_2.5_ sites and 112 NO_2_ sites across a study region of approximately 1.57 million km^2^, whereas the ELAPSE European study^25^ modeled data for 543 PM_2.5_ sites and 2399 NO_2_ sites, mainly within cities across western Europe (total land area not specified), with external validation conducted on 416 and 1396 sites, respectively, and two US studies used data from 2156 PM_2.5_ sites^22^ and 912 NO_2_ sites^23^ across the contiguous US continent with an area of 8.08 million km^2^. Given the limitation of a sparse monitoring network for much of our study area, we had anticipated that more sophisticated ensemble modeling might have added value and yielded predictions with significantly better precision than the base ML models tested; however this was not the case. One explanatory factor might be the comparatively small concentration ranges of our monitored pollutants, and thus smaller variation of outcome data in our models. It will be interesting for future research to determine whether ensemble methods meaningfully outperform base ML models when applied in low- and middle-income regions, which also suffer from low density monitoring networks, but which experience a much greater pollutant concentration range. A case in point is a PM_10_ modeling study developed for four sparsely monitored regions in South Africa (total modeling domain area unspecified with data from 18 monitors) which reported lower, but similar, performance of the ensemble model (total CV *R* ^2^ = 0.81 RMSE = 11.4 μg/m^3^) compared to a base learner RF model.^72^

The finding of only modest improvement of the ensemble model over the base ML models, also suggests, that at least in our setting, when assessed using random CV, the base ML models performed well and captured almost as much variation as the ensemble model with minimal loss of precision (reflected in the RMSEs), a finding also seen in studies elsewhere for PM_2.5_ ^22,27,29^ and NO_2_.^23,25^ In Xiao et al.’s (2018) comparison of an ensemble model with four ML model inputs including XGBoost, RF and generalized additive models, which predicted historical daily PM_2.5_ across China, the ensemble model *R* ^2^ and RMSE outputs were almost identical (*R* ^2^ = 0.85 (RMSE = 18 μg/m^3^) (CV *R* ^2^ = 0.79)) with the XGBoost model outputs (*R* ^2^ = 0.84 (RMSE = 18 μg/m^3^) (CV *R* ^2^ = 0.78)). Chen et al., 2019, reported that an ensemble “stacking” model had a marginally higher *R* ^2^ and lower RMSE (*R* ^2^ = 0.85 (RMSE = 17.3 μg/m^3^)) compared with the best ML models: XGBoost (*R* ^2^ = 0.84 (RMSE = 18.1 μg/m^3^)), Adaboost (*R* ^2^ = 0.84 (RMSE = 18.3 μg/m^3^)), and a RF model (*R* ^2^ = 0.82 (RMSE = 19.6 μg/m^3^)), in predicting hourly PM_2.5_ concentrations for China using satellite data. Similarly, two North American studies using the same methodology^22,23^ demonstrated slightly better performance from an ensemble model used to predict daily PM_2.5_ ^22^ and NO_2_ ^23^ on a 1 km grid across the contiguous US, with overall CV *R* ^2^ values only slightly higher than the ML models tested (RF, gradient boosting, and neural networks). However, Di et al. (2019) also noted the ensemble model with a spline fit between predicted and measured daily PM_2.5_ was almost linear up to a concentration of 60 μg/m^3^, demonstrating good performance at high PM_2.5_ concentrations, whereas the RF and GB models overestimated at high concentrations. They further highlighted that the three ML models could be considered as complementary as no single model was found to perform equally well for each US region. Similarly, Di et al.’s^23^ NO_2_ study showed good linearity for their ensemble model up to high annual average concentrations of 55 ppb. In contrast, the ELAPSE study, reporting on test results from 16 algorithms using external validation data sets for prediction of annual average PM_2.5_ and NO_2_ concentrations across Europe, found that some of the base learner ML models performed better than the ensemble models for PM_2.5_, but not for NO_2_.^25^

The model fit for our PM_10_ and NO_2_ models (random CV *R* ^2^ and test *R* ^2^ and RMSEs) for both the ensemble (DEML) and best performing base ML models were on par with model statistics for comparably validated ensemble and ML models reported elsewhere from Europe^25^ and North America.^23^ This provides confidence in our resultant pollutant predictions despite monitoring in a relatively low-density monitor setting. However, for PM_2.5_, studies elsewhere that have compared ensemble models with ML models in the US,^22^ Italy,^26^ and China,^27,29,30^ all reported substantially better model fit (*R* ^2^) than our DEML model. The likely reasons for this are the greater number and geographic spread of monitored sites and the greater range in PM_2.5_ concentrations in the overseas regions studied. For instance in this study we reported a mean concentration for PM_2.5_ in NSW for the period 2005–2018 of 6.9 μg/m^3^ (range 5.7–7.5 μg/m^3^) compared to a mean of 77.4 μg/m^3^ (range: 5–477 μg/m^3^; SD: 68.4 μg/m^3^) for Beijing,^30^ and means of 16–18 μg/m^3^ (range: approximately 2–25 μg/m^3^) in Italy.^26^ A strength of our study is the use of a hold-out test data set used for validation in addition to normal CV methods.^73^ Our test statistics showed that, when the model was fit to the test data, the overall model fit decreased and prediction error increased compared with the CV model, with changes in *R* ^2^ and RMSEs as expected. However, we acknowledge that the random CV method used by us maximizes the amount of data that is able to be used to train the models. Accordingly, our performance measures, namely *R* ^2^ and RMSE, for both the training and test data sets, are likely to be overoptimistic in comparison to overseas models where spatial CVs have been reported. However, it is important to note that some researchers argue that random CV can be particularly useful when available data are more limited, and that relying solely on spatial CV can result in performance measures which are overly pessimistic.^70,71^

Nevertheless, the results from our spatial CVs for our base ML models and second-stage meta-learner ML models indicated a substantial decrease in *R* ^2^ and increase in RMSE, a finding that has also been reported elsewhere, from studies with both large and small sample sizes. In the European ELAPSE study,^25^ the best performing base ML models for PM_2.5_ (RF, Bagging, GBM, *R* ^2^ ranging from 0.895 to 0.95), experienced a substantial drop in model fit when tested with a held-out validation data set (*R* ^2^ ranged from 0.626 to 0.631) and spatial CV (*R* ^2^ ranged from 0.530 to 0.548). The European study results for NO_2_ showed similar reductions between CV models and models fit with test data, suggesting that the CV models had a limited ability to generalize beyond their training domain, possibly due to spatial or temporal extrapolation challenges. Modeling of PM_10_ in South Africa also reported substantially reduced *R* ^2^ for their spatial CV models, although their spatial CV *R* ^2^ = 0.48 for the ensemble model was higher than for the learner ML models.^72^ In contrast, two US studies reported higher performance for their ensemble and base learner spatial CV models compared to the overall CV models for NO_2_ ^23^ and PM_2.5_,^22^ which might be attributable to the large number of and good geographic coverage of monitoring sites across the contiguous US. However, they also reported greater uncertainty in the performance of the NO_2_ ensemble model for areas with sparser monitoring.^23^

Model prediction error along with model fit (*R* ^2^) is a useful measure in judging model performance, with some stating that it is more relevant for estimating health impacts.^73^ This is because the *R* ^2^ can vary depending on the spatiotemporal domain, with a larger domain often leading to model improvement, but not necessarily greater precision.^73^ The RMSEs for all of the pollutants in our study were low (1.07 μg/m^3^, 1.90 μg/m^3^ and 1.18 ppb for PM_2.5_, PM_10_ and NO_2_ respectively) compared with most other studies cited above. This suggests good precision of estimates in our study, although the low RMSEs are also likely to reflect the much smaller range in pollutant concentrations experienced in Australia compared with other regions, especially for annual mean predictions.

Among the base learner models in our study, the SLR model, based on the linear regression approach, performed the weakest, resulting in the lowest *R* ^2^ and highest RMSEs for both the CV and test models. This is likely due to their linear function which is inherently unable to capture the complex, and potentially nonlinear, relationships between pollutant concentrations and the underlying predictors.^12^ In addition, the supervised approach to variable selection resulted in a lower number of predictor variables used in the SLR models (*n* = 9 for NO_2_; *n* = 13 for PM_2.5_), compared with the ML models which incorporated 79 predictor variables of heterogeneous nature. Other studies have similarly reported improved model fit for ML models compared with linear regression models,^25,28,74^ geostatistical models,^75,76^ and hybrid interpolation models.^76^ In contrast, a study which modeled PM_2.5_ across the contiguous US reported poorer performance of ML models compared with the geo-statistical models tested, which the authors attributed to the smaller number of variables used in the ML models and suggested that the geostatistical models better captured spatial relationships.^64^ Taiwanese researchers have reported that combining SLR (LUR) models with ML models such as RF, deep neural networks, and XG Boost resulted in better performance for PM_2.5_ prediction.^65^

We determined the major predictors of influence for the best-performing base models for each pollutant. For the PM_2.5_ XGBoost model, the major predictors were related to meteorological and geographic conditions (i.e., seasonal winter mean daily solar exposure (13.3%), maximum yearly average temperature (3.8%), annual rainfall (2.5%), and latitude (8.9%)); measurement type (use of a BAM TEOM for measurement (7.4%)); tree cover within 10km^2^ (5.1%); percentage of burned area within 100 km (3.6%); NPI emissions of PM_2.5_ within 10 km (2.8%); residential land use within 100 m (2.4%), and satellite estimated NO_2_ (2.3%). These are commonly identified as PM_2.5_ predictors. Meteorology is a well-known driver of PM_2.5_ concentrations globally, including in the Sydney region, where a PM_2.5_ source apportionment study found that meteorological influences played a major part in PM_2.5_ variability, accounting for 41% of total PM_2.5_ ^77^. The same study also reported that temperature impacted significantly on total PM_2.5._ Predictors related to natural sources such as tree cover and degree of burned area are consistent with a previous NSW modeling study,^78^ which reported that natural sources such as biogenic emissions, sea salt, and wind-blown dust contribute approximately 30–60% of total PM_2.5_ across the GSA, and a combined modeling and measurement study, the Sydney Particle Study,^79^ which reported that volatile organic chemicals released from vegetation were a major source of secondary organic aerosols in the summer months. The predictors related to NPI PM_2.5_ emissions, residential land use, and satellite estimated NO_2_ are all associated with anthropogenic sources and activity.

For the PM_10_ GBM model, the major predictors were: year of measurement (22.6%); NPI emissions of PM_2.5_ (5.8% and 5.5%) and NOx (2.9% and 2.3%) within 5 and 10 km, respectively; satellite estimated NO_2_ (5%); elevation (4.5%); tree cover within 10 km (2.2%), average building height within 100 m (2.1%); and park land use within 10 km (1.9%). These influential predictors are not surprising, with year of measurement having the greatest influence. In NSW, government monitoring has shown that exceedances of PM_10_ in NSW can vary by year depending on the extent and location of wildfires, fire hazard reduction burns, agricultural activity, dust storms, and industrial sources in the Hunter region.^80^ Consistent with this finding, Table S5 shows that peak PM_10_ concentrations occurred in 2009, corresponding to a drought period in NSW and an increasing occurrence of dust storms.^80^ The predictors related to NPI emissions and satellite estimated NO_2_ are consistent with secondary aerosols being a substantial contributor to PM_10_ in general, and the aforementioned industrial sources (particularly coal mining), with government reporting noting that industry accounted for 30–69% of total PM_10_ emissions in 2008 across various urban and nonurban NSW regions.^81^

For the GBM NO_2_ model, the predictors of major influence were: population density within 10 km (>25%); satellite estimated NO_2_ (8.4%); NPI estimated PM_2.5_ within 10 km (7.9%); year of monitoring (6.2%); average building height within 100 m (5.1%); and various traffic related variables such as major road length within 200 m (5.7%), distance to roads (4.4%), and minor road length within 10 km (4.2%). Most of these predictors have been reported in previous modeling of NO_2_. Population density can be viewed as a proxy indicator of vehicle density and use, human movement and activity, and level of urbanization, and was the most influential predictor at 25.6%. Unsurprisingly, satellite estimated NO_2_ (8.4%) was the second most influential predictor, although our finding was in contrast to Di et al.’s^23^ finding where satellite derived column estimates contributed <1% to various base ML models. NPI estimates of PM_2.5_ within 10 km is suggestive of industrial processes that may be correlated with increased NOx emissions, a precursor to NO_2._ Traffic exhaust is a known major contributor to NOx emissions in the GSA^82^ and traffic related variables such as traffic density/counts, density of major roads, and distance to roads, are well-known predictors of NO_2_ in previous models.^55,83^ The year of NO_2_ measurement, another major influential predictor, is consistent with the gradual declines in NO_2_ reported in Table S6 which show peaks in mean, median, and 75% percentile annual concentrations in 2006 and 2008 and then a very gradual decline to 2018.

We note the difference in spatial variation between the ensemble modeled PM_2.5_ and PM_10_ maps and hypothesize that this is due to the majority of PM_10_ in western NSW being associated with crustal and agricultural sources whereas PM_2.5_ variation may more strongly reflect traffic sources in these regions where background PM_2.5_ concentrations are very low. The low modeled background PM_2.5_ concentrations in rural areas is consistent with results from the government rural network of Dustwatch monitors which use DustTraks to measure PM_2.5_, and which measured an annual mean for 2023 of 2.3 μg/m^3^ (range: 1 to 5.9 μg/m^3^), and for 2024 a mean of 2.3 μg/m^3^ (range: 1 to 4.2 μg/m^3^). It is possible that the PM_2.5_ model has overemphasized the relationship between the road network and PM_2.5_, given that traffic related variables were not among the major influential predictors in the XGBoost base model. However, in NSW urban settings, a number of source-apportionment, monitoring and modeling studies have highlighted traffic vehicles as a source of PM_2.5_.^77,78,84,85^ Furthermore, a case study which measured PM_2.5_ along and away from a main road in Sydney found that roadside PM_2.5_ concentrations were twice those of a background monitoring site, and that concentrations reduced by almost 30% within 50 m from the road.^85^ Given the very low PM_2.5_ concentrations in much of rural NSW, it is not incongruous that traffic exhaust, especially from diesel related heavy vehicles, might contribute disproportionately to PM_2.5_ concentrations and so reflect roadside conditions, as portrayed in Figure 6a. The PM_10_ maps predict the highest concentrations in an area north of Sydney, the Hunter Region. This might be explained by the location of several large-scale coal mines and coal-fired power stations in the area and is consistent with the main variables of influence for the PM_10_ model being those related to the NPI, as mentioned above, and is also consistent with previous government modeling showing higher PM_10_ concentrations in this area.^81^

From an epidemiological perspective, the priority in testing and comparing various modeled exposure surfaces is to reduce the error in the exposure assignment, so as to minimize attenuation or bias in exposure-response function estimates.^86,87^ Therefore, much research effort is often expended on refining exposure modeling. However, from a pragmatic viewpoint, the choice of modeling procedure is often a trade-off between a number of factors. One of these is the availability of resources including people, data, and computing (storage, memory, resampling).^11^ Another factor relates to the purpose of the model. For instance, the difference where the purpose of the model is to ensure that predictors and the direction of effect are interpretable (e.g., to inform policy changes)^12^ compared to a model that is used in an “estimative” capacity. With the latter, the objective is to produce the most precise predictions possible without needing to rationalize or explain the inputs or predictors.

In our study, the DEML model fit the data the best; however, the model fit and precision were only slightly improved over the base learner ML models for PM_10_ and NO_2_. However, for PM_2.5_, the model fit of the DEML model was similar to that of the best meta-learner (2nd stage) model, the GLMNET model. The modest improvement of the ensemble models is notable given our low-density monitoring network, smaller number of monitoring stations, and our low pollution setting when compared internationally. However, like studies conducted elsewhere, we found that for our setting, the type of cross-validation, that is, random, held-out, or spatial CV, had a substantial impact on the model performance. We suggest that the true model performance is likely to lie between the more optimistic test results reported for the random CV models and the more pessimistic test results that were reported for the spatial CV models. While we support the development of the most robust exposure models possible for use in exposure-response studies, the modest improvement of the DEML model framework over the base and meta-learner ML models suggests the need to balance the choice of modeling framework and model implementation with the data and project resources available along with consideration of the constraints associated with the local setting.

## Supplementary Material



## Notes

ESCAPE Protocols; Hyperparameters of base learner models; hyperparameters of base learner ML algorithms; details of candidate predictor variables; GIS data used as predictor variables; GIS variable definitions; definitions of predictor variables; descriptive statistics for pollutants monitored by government and project monitors; descriptive statistics for annual average PM_2.5_ (μg/m^3^); descriptive statistics for annual average PM_10_ (μg/m^3^); descriptive statistics for annual average NO_2_ (ppb); spatial CV results for base learner and meta-learner evaluations; spatial cross-validation (CV) results for base learner and meta-learner evaluations for PM_2.5_, PM_10_, and NO_2_; relative influence of predictors for best performing base-learner models for prediction of PM_2.5_, PM_10_, and NO_2_ (refer to Table S2 and S3 for variable definitions); relative influence of XGBoost base-learner (best performing base learner ML model) for PM_2.5_ prediction; relative influence of GBM base-learner (best performing base learner ML model) for PM_10_ prediction; and relative influence of GBM base-learner (best performing base learner ML model) for NO_2_ prediction (PDF)
