# Supplementary material for: Comparison of High Spatial Resolution PM2.5, PM10, and NO2 Estimates Using a Deep Ensemble Machine Learning Framework in a Low Pollution Setting
Source: Environ Sci Technol. 2026 Jun 30;60(27):19449–64. doi: 10.1021/acs.est.6c02076 (PMC13374088; doi:10.1021/acs.est.6c02076)

Supporting Information

Comparison of high spatial resolution $PM_{2.5}$, PM_{10} and NO_2 estimates using a deep ensemble machine learning framework in a low pollution setting

Christine T Cowie*[a,b,m], Ivan C Hanigan[c,m,o], Wenhua Yu[d], Cassandra Yuen[e], Karthik Gopi[e,o], Geoffrey G Morgan[e,m,o], Nicolas Borchers-Arriagada[f,m], Jane Heyworth[g,m], Martin Cope[h,m], Lidia Morawska[i,m,n], Bin Jalaludin[j,m], Guy B Marks[a,b,k,m], Yuming Guo[d,m], Luke D Knibbs[l,m]

[a] Woolcock Institute of Medical Research, Macquarie University
Macquarie Park, NSW 2113, Australia
[b] South West Sydney Clinical Campus, UNSW Sydney, Liverpool NSW 2170, Australia
[c] School of Population Health, Curtin University, Perth, Western Australia, 6845, Australia
[d] School of Public Health and Preventive Medicine, Monash University, Melbourne, VIC 3004, Australia
[e] Sydney School of Public Health, and University Centre for Rural Health, The University of Sydney, Sydney, NSW 2050, Australia
[f] Menzies Institute for Medical Research, University of Tasmania, Hobart, TAS 7000, Australia
[g] The School of Population and Global Health, The University of Western Australia, Perth, Crawley WA 6009, Australia
[h] Commonwealth Scientific, Industrial & Research Organisation (CSIRO), Aspendale, VIC 3195, Australia
[i] International Laboratory for Air Quality and Heath (ILAQH), School of Earth & Atmospheric Sciences, Queensland University of Technology, Brisbane, QLD 4000, Australia
[j] School of Population Health, University of NSW, Sydney, NSW 2052, Australia
[k] Burnet Institute, Melbourne, Victoria 3001, Australia
[l] Public Health Research Analytics and Methods for Evidence, Sydney Local Health District, Camperdown NSW 1450, Australia
[m] Centre for Safe Air, NHMRC Centre for Research Excellence, Hobart, TAS 7000, Australia
[n] Global Centre for Clean Air Research (GCARE), University of Surrey, Guildford GU2 7XH, UK
[o] Healthy Environments And Lives (HEAL) National Research Network, Canberra, ACT, 2617, Australia

*christine.cowie@macquarie.edu.au

Supporting Information

ESCAPE Protocols S3

Hyperparameters of base learner models
Table S1: Hyperparameters of base learner machine learning algorithms S3

Details of candidate predictor variables
Table S2: GIS data used as predictor variables S4

GIS variable definitions
Table S3: Definitions of predictor variables S12

Descriptive statistics for pollutants monitored by government & project monitors*
Table S4 Descriptive statistics for annual average $PM_{2.5}$ ($\mu g/m^3$) S17
Table S5 Descriptive statistics for annual average PM_{10} ($\mu g/m^3$) S17
Table S6 Descriptive statistics for annual average NO_2 (ppb) S18

Spatial Cross validation (CV) results for base learner & meta-learner evaluations
Table S7 Spatial cross-validation (CV) results for base learner and meta-learner evaluations for $PM_{2.5}$, PM_{10}, and NO_2 S19

Relative influence of predictors for best performing base-learner models for prediction of $PM_{2.5}$, PM_{10} and NO_2 (refer to Table S2 and S3 for variable definitions)
Figure S1-Relative influence of XGBoost base-learner (best performing base learner ML model) for $PM_{2.5}$ prediction S20
Figure S2-Relative influence of GBM base-learner (best performing base learner ML model) for PM_{10} prediction S21
Figure S3-Relative influence of GBM base-learner (best performing base learner ML model) for NO_2 prediction S22

Supplementary information

ESCAPE Protocols

ESCAPE Study. SOP ESCAPE 1.0. Measurement of $PM_{2.5}$ and PM_{10} in outdoor air with the Harvard Impactor. Institute of Risk Assessment Sciences (IRAS), University of Utrecht, 2008.

ESCAPE Study. SOP ESCAPE 2.0 V1. Measurement of NO_2 and NO_x in outdoor air with the Ogawa Badge, Institute for Risk Assessment Sciences (IRAS), Utrecht University, 2009.

ESCAPE Study. ESCAPE Exposure Assessment Manual, Version 9. Formerly available at: www.escapeproject.eu, 2010.

Hyperparameters of base learner models

Table S1: Hyperparameters of base learner machine learning algorithms

Model	Hyperparameter	Value
Random Forest	mtry	3
	min.node.size	5
XGBoost	eta	0.1
	min_child_weight	10
	max_depth	4
GBM	shrinkage	0.1
	n.trees	10000
	interaction.depth	3
	n.minobsinnode	20

Details of candidate predictor variables

Table S2: GIS data used as predictor variables

Predictor type	Variable_name	Project ID	Dataset ID	Citation
Response variable	no2_annual	Air_Pollution_Monitoring_Stations_National_RESTRICTED	CARs_National_Air_Pollution_Database	Centre for Air pollution, energy and health Research, 2021. National Air Pollution Monitoring Database, derived from regulatory monitor data from NSW DPIE, Vic EPA, Qld DES, SA EPA, WA DEWR, Tas EPA, NT EPA, and ACT Health. Downloaded from the Centre for Air pollution, energy and health Research [accessed 2021-03-30] DOI 10.17605/OSF.IO/JXD98
Response variable	pm25_annual	Air_Pollution_Monitoring_Stations_National_RESTRICTED	CARs_National_Air_Pollution_Database	Centre for Air pollution, energy and health Research, 2021. National Air Pollution Monitoring Database, derived from regulatory monitor data from NSW DPIE, Vic EPA, Qld DES, SA EPA, WA DEWR, Tas EPA, NT EPA, and ACT Health. Downloaded from the Centre for Air pollution, energy and health Research [accessed 2021-05-04] DOI 10.17605/OSF.IO/JXD98
Response variable	pm10_annual	Air_Pollution_Monitoring_Stations_National_RESTRICTED	CARs_National_Air_Pollution_Database	Centre for Air pollution, energy and health Research, 2021. National Air Pollution Monitoring Database, derived from regulatory monitor data from NSW DPIE, Vic EPA, Qld DES, SA EPA, WA DEWR, Tas EPA, NT EPA, and ACT Health. Downloaded from the Centre for Air pollution, energy and health Research [accessed 2021-05-21] DOI 10.17605/OSF.IO/JXD98
Satellite-based air pollution estimates	sat_no2	Satellite_OMI_NO2	Sat_OMI_NO2_LK_2005_2019	Nickolay A. Krotkov, Lok N. Lamsal, Sergey V. Marchenko, Edward A. Celarier, Eric J.Bucsela, William H. Swartz, Joanna Joiner and the OMI core team (2019), OMI/Aura NO2 Cloud-Screened Total and Tropospheric Column L3 Global Gridded 0.25 degree x 0.25 degree V3, NASA Goddard Space Flight Center, Goddard Earth Sciences Data and Information Services Center (GES DISC), Accessed: mid 2020, 10.5067/Aura/OMI/DATA3007 Downloaded from the Centre for Air pollution, energy and health Research https://cloud.car-dat.org/index.php/apps/files/?dir=/ResearchProjects_CAR/Satellite_OMI_NO2/Sat_OMI_NO2_LK_2005_2019
Satellite-based air pollution estimates	sat_pm25	Atmospheric_Composition_Analysis_Group	annual_surface_pm25_concentrations_2005_2018	Atmospheric Composition Analysis Group, 2020. Estimated global annual surface fine particulate matter (PM2.5) concentrations for other regions, 2005-2018. Available from https://sites.wustl.edu/acag/datasets/surface-pm2-5/ [accessed late 2020]. Downloaded from the Centre for Air pollution, energy and health Research https://cloud.car-

Predictor type	Variable_name	Project ID	Dataset ID	Citation
				dat.org/index.php/apps/files/?dir=/Environment_General/Atmospheric_Composition_Analysis_Group/annual_surface_pm25_concentrations_2005_2018
Satellite-based air pollution estimates	sat_o3	TEMIS_Tropospheric_Emission	O3_Multi_Sensor_Reanalysis_MSR2_1979_2019	Van der A, R. J., Allaart, M. A. F., and Eskes, H. J., (2015): Multi-Sensor Reanalysis (MSR) of total ozone, version 2. Royal Netherlands Meteorological Institute (KNMI). (Dataset). doi: 10.21944/temis-ozone-msr2. Downloaded from the Centre for Air pollution, energy and health Research https://cloud.car-dat.org/index.php/apps/files/?dir=/CAR_staging_area/TEMIS_Tropospheric_Emission/O3_Multi_Sensor_Reanalysis_MSR2_1979_2019
Temporal	year	Air_pollution_modelling_APMMA	AP_monitor_locations	Centre for Air pollution, energy and health Research, 2021. State and Territory government regulatory air pollution monitor locations provided by NSW DPIE, ACT Health, SA EPA, Qld DES, Vic EPA (manually checked to correct for minor discrepancies), combined with field monitor locations from Ogawa Patches and Harvard Impactors at various Sydney GMR regions.
Position and Elevation	lon_metres	Air_pollution_modelling_APMMA	AP_monitor_locations	
Position and Elevation	lat_metres	Air_pollution_modelling_APMMA	AP_monitor_locations	
Position and Elevation	state	Air_pollution_modelling_APMMA	AP_monitor_locations	
Position and Elevation	pos_elevation	Digital_Elevation_Model_GA	SRTM_1sec_Smooth_DEM_National	Gallant, J., Wilson, N., Dowling, T., Read, A., Inskeep, C. 2011. SRTM-derived 1 Second Digital Elevation Models Version 1.0. Record 1. Geoscience Australia, Canberra. http://pid.geoscience.gov.au/dataset/ga/72759
Position and Elevation	pos_distocean	Water_bodies_GA	Australian_maritime_boundaries_2014	Alcock, M.B., McGregor, M.J., Hatfield, A, Taffs, N.J. (2014): Seas and Submerged Lands Act 1973 - Australian Maritime Boundaries 2014a - Geodatabase. Geoscience Australia, Canberra. (Dataset). http://dx.doi.org/10.4225/25/5539DFE87D895. Downloaded from the Centre for Air pollution, energy and health Research https://cloud.car-dat.org/index.php/apps/files/?dir=/Environment_General/Water_bodies_GA/Australian_maritime_boundaries_2014
Monitor properties	monitor_type	Air_pollution_modelling_APMMA	AP_monitor_locations	Centre for Air pollution, energy and health Research, 2021. State and Territory government regulatory air pollution monitor locations provided by NSW DPIE, ACT Health, SA EPA, Qld DES, Vic EPA (manually checked to correct for minor discrepancies), combined with field monitor locations from Ogawa Patches and Harvard Impactors at various Sydney GMR regions.

Predictor type	Variable_name	Project ID	Dataset ID	Citation
Monitor properties	bam_teom	Air_pollution_modelling_APMMA	AP_monitor_locations	Centre for Air pollution, energy and health Research, 2021. State and Territory government regulatory air pollution monitor locations provided by NSW DPIE, ACT Health, SA EPA, Qld DES, Vic EPA (manually checked to correct for minor discrepancies), combined with field monitor locations from Ogawa Patches and Harvard Impactors at various Sydney GMR regions.
Land use	lu_com_{buffer}	ABS_customised_for_APMMA	apmma_abs_mb	Centre for Air pollution, energy and health Research, 2021. ABS Meshblock data re-coded by for the APMMA GIS air pollution modelling project. Restricted.
Land use	lu_res_{buffer}	ABS_customised_for_APMMA	apmma_abs_mb	Centre for Air pollution, energy and health Research, 2021. ABS Meshblock data re-coded by for the APMMA GIS air pollution modelling project. Restricted.
Land use	lu_open_{buffer}	ABS_customised_for_APMMA	apmma_abs_mb	Centre for Air pollution, energy and health Research, 2021. ABS Meshblock data re-coded by for the APMMA GIS air pollution modelling project. Restricted.
Land use	lu_ind_{buffer}	ABS_customised_for_APMMA	apmma_abs_mb	Centre for Air pollution, energy and health Research, 2021. ABS Meshblock data re-coded by for the APMMA GIS air pollution modelling project. Restricted.
Land use	lu_bldg_hgt_avg_100	Geoscape	Geoscape Residential land	PSMA, 2019-08-12, Geoscape_2019, [Data set] Retrieved from CARDAT
Land use	lu_bldg_foot_area_100	Geoscape	Geoscape Residential land	PSMA, 2019-08-12, Geoscape_2019, [Data set] Retrieved from CARDAT
Land cover	lc_tree_cover_{buffer}	Land_Cover_MODIS	tree_and_water_coverage_2017_2018	DiMiceli, C., Carroll, M., Sohlberg, R., Kim, D., Kelly, M., Townshend, J. (2015). MOD44B MODIS/Terra Vegetation Continuous Fields Yearly L3 Global 250m SIN Grid V006 [Data set]. NASA EOSDIS Land Processes DAAC. Accessed 2021-05-10 from https://doi.org/10.5067/MODIS/MOD44B.006 (Terra Product ID 250m MOD44B v6 march 2017-march 2018). Available through https://modis.gsfc.nasa.gov/data/dataprod/mod44.php
Land cover	lc_park_{buffer}	ABS_customised_for_APMMA	apmma_abs_mb	Centre for Air pollution, energy and health Research, 2021. ABS Meshblock data re-coded by for the APMMA GIS air pollution modelling project.
Land cover	lc_water_modis_{buffer}	Land_Cover_MODIS	tree_and_water_coverage_2017_2018	DiMiceli, C., Carroll, M., Sohlberg, R., Kim, D., Kelly, M., Townshend, J. (2015). MOD44B MODIS/Terra Vegetation Continuous Fields Yearly L3 Global 250m SIN Grid V006 [Data set]. NASA EOSDIS Land Processes DAAC. Accessed 2021-05-10 from https://doi.org/10.5067/MODIS/MOD44B.006 (Terra Product ID 250m MOD44B v6 march 2017-march 2018).

Predictor type	Variable_name	Project ID	Dataset ID	Citation
				Available through https://modis.gsfc.nasa.gov/data/dataprod/mod44.php
Land cover	lc_impsa_{buffer}	Landsat_Global_High_Resolution_Urban_Data	Landsat_GMIS_v1_Australia_2010	Brown de Colstoun, E. C., C. Huang, P. Wang, J. C. Tilton, B. Tan, J. Phillips, S. Niemczura, P.-Y. Ling, and R. E. Wolfe. 2017. Global Man-made Impervious Surface (GMIS) Dataset From Landsat. Palisades, New York: NASA Socioeconomic Data and Applications Center (SEDAC). https://doi.org/10.7927/H4P55KKF. Downloaded from the Centre for Air pollution, health and energy Research https://cloud.car-dat.org/index.php/apps/files/?dir=/Environment_General/Landsat_Global_High_Resolution_Urban_Data/Landsat_GMIS_v1_Australia_2010
Emission sources	em_minrd_invdist	Traffic_Load	Traffic_Load_Australia_2018	Yuen, C, 2020. Traffic load intensity derived from Zenith 2018 traffic counts and PSMA 2019 road networks. Downloaded from the Centre for Air pollution, health and energy Research https://cloud.car-dat.org/index.php/apps/files/?dir=/ResearchProjects_CAR/Traffic_Load/Traffic_Load_Australia_2018
Emission sources	em_majrd_invdist	Traffic_Load	Traffic_Load_Australia_2018	
Emission sources	em_majrd_length_{buffer}	Traffic_Load	Traffic_Load_Australia_2018	
Emission sources	em_minrd_length_{buffer}	Traffic_Load	Traffic_Load_Australia_2018	
Emission sources	em_rds_dist	Traffic_Load	Traffic_Load_Australia_2018	
Emission sources	em_rds_int_imputed	Traffic_Load	Traffic_Load_Australia_2018	
Emission sources	em_rds_intinvdist_imputed	Traffic_Load	Traffic_Load_Australia_2018	
Emission sources	em_all_rds_intinvdist_imputed	Traffic_Load	Traffic_Load_Australia_2018	
Emission sources	em_all_rds_int_imputed	Traffic_Load	Traffic_Load_Australia_2018	
Emission sources	em_wood_heat_pct	Woodfire_heaters	household_woodfire_heaters_Australia_2012	Knibbs, 2021. Household woodfire heater data, derived from ABS survey data on primary household energy source 2012. Downloaded from the Centre for Air pollution, energy and health research https://cloud.car-dat.org/index.php/apps/files/?dir=/Environment_General/Woodfire_heaters/household_woodfire_heaters_Australia_2012
Emission sources	em_burned_area_pct_{buffer}	Fire_MODIS	NASA_MODIS_burnt_area_2005_2019	Giglio, L., Schroeder, W., Hall, J.V., & Justice, C.O., 2020. Collection 6 MODIS MCD64 Burned Area Product, data derived from NASA MODIS data. Available from

Predictor type	Variable_name	Project ID	Dataset ID	Citation
				https://modis-fire.umd.edu/index.html. Downloaded from the Centre for Air pollution, energy, and health Research https://cloud.car-dat.org/index.php/apps/files/?dir=/Environment_General/Fire_MODIS/NASA_MODIS_burnt_area_2005_2019
Emission sources	em_fire_dens_fireskm2_{buffer}	Fire_MODIS	NASA_MODIS_active_fires_2005_2015<br>NASA_MODIS_active_fires_2016_2019	Giglio, L., Schroeder, W., Hall, J.V., & Justice, C.O. (2021): Collection 6 MOD14CMQ and MYD14CMQ monthly active fire climate modeling grid (CMG) 2005-2015. NASA MODIS. (Dataset). https://modis-fire.umd.edu/index.html. Downloaded from Centre for Air pollution, energy, and health Research https://cloud.car-dat.org/index.php/apps/files/?dir=/Environment_General/Fire_MODIS/NASA_MODIS_active_fires_2005_2015<br>Giglio, L., Schroeder, W., Hall, J.V., & Justice, C.O., 2020. Collection 6 MOD14CMQ and MYD14CMQ monthly active fire climate modeling grid (CMG) data derived from NASA MODIS data 2016-2019. Available from https://modis-fire.umd.edu/index.html. Downloaded from the Centre for Air pollution, energy, and health Research https://cloud.car-dat.org/index.php/apps/files/?dir=/Environment_General/Fire_MODIS/NASA_MODIS_active_fires_2016_2019
Emission sources	em_npi_{pollutant}_dens_siteskm2{buffer}	NPI	NPI_NOX_Australia_2017_2018<br>NPI_PM25_Australia_2010_2011	Department of Environment and Energy, 2021. National Pollutant Inventory point source air-based emissions for NOx Australia 2017-2018. Australian Government. Downloaded from the Centre for Air pollution, energy and health Research https://cloud.car-dat.org/index.php/apps/files/?dir=/Environment_General/NPI/NPI_NOX_Australia_2017_2018
Emission sources	em_npi_{pollutant}_dens_kgkm2{buffer}			Department of Environment and Energy, 2021. National Pollutant Inventory point source air-based emissions for PM2.5 Australia 2010-2011. Australian Government. Downloaded from the Centre for Air pollution, energy and health Research https://cloud.car-dat.org/index.php/apps/files/?dir=/Environment_General/NPI/NPI_PM25_Australia_2010_2011
Population	pop_dens_{buffer}	ABS_data	ABS1x1km_Aus_Pop_Grid_2006_2020	Australian Bureau of Statistics (2021). Regional Population Growth, Australia, 2006-2020, cat. no. 3218.0 https://www.abs.gov.au/ausstats/abs@.nsf/mf/3218.0. Retrieved from the Centre for Air pollution, energy and health Research https://cloud.car-dat.org/index.php/apps/files/?dir=/Environment_General/ABS_data/ABS1x1km_Aus_Pop_Grid_2006_2020

Predictor type	Variable_name	Project ID	Dataset ID	Citation
Weather	wthr_maxtmp_yrly_avg	AWAP_GRIDS	AWAP_GRIDS_monthly_temperature_1986_2019	Australian Bureau of Meteorology, Bureau of Rural Sciences, and CSIRO (2021). Australian Water Availability Project (AWAP) Temperature Grid Data aggregated to month 1986-2019. Data retrieved from Centre for Air pollution, energy and health Research https://cloud.car-dat.org/index.php/apps/files/?dir=/Environment_General/AWAP_GRIDS/AWAP_GRIDS_monthly_temperature_1986_2019
Weather	wthr_meantmp_yrly_avg	AWAP_GRIDS	AWAP_GRIDS_monthly_temperature_1986_2019	Australian Bureau of Meteorology, Bureau of Rural Sciences, and CSIRO (2021). Australian Water Availability Project (AWAP) Temperature Grid Data aggregated to month 1986-2019. Data retrieved from Centre for Air pollution, energy and health Research https://cloud.car-dat.org/index.php/apps/files/?dir=/Environment_General/AWAP_GRIDS/AWAP_GRIDS_monthly_temperature_1986_2019
Weather	wthr_drt_count_months	DROUGHT-AWAP-GRIDS-RESTRICTED	Drought_and_APMMA_locations_2005_2019	Hanigan, I. & Van Buskirk, J. 2021. AWAP SPI and SPEI drought indices and duration at APMMA locations 2005-2019. Data downloaded from the Centre for Air pollution, energy and health Research https://cloud.car-dat.org/index.php/apps/files/?dir=/ResearchProjects_CAR/DROUGHT-AWAP-GRIDS-RESTRICTED/Drought_and_APMMA_locations_2005_2019
Weather	wthr_drt_max_length	DROUGHT-AWAP-GRIDS-RESTRICTED	Drought_and_APMMA_locations_2005_2019	Hanigan, I. & Van Buskirk, J. 2021. AWAP SPI and SPEI drought indices and duration at APMMA locations 2005-2019. Data downloaded from the Centre for Air pollution, energy and health Research https://cloud.car-dat.org/index.php/apps/files/?dir=/ResearchProjects_CAR/DROUGHT-AWAP-GRIDS-RESTRICTED/Drought_and_APMMA_locations_2005_2019
Weather	wthr_rain_yrly_total	AWAP_GRIDS	AWAP_GRIDS_monthly_rainfall_1900_2020	Australian Bureau of Meteorology, Bureau of Rural Sciences, and CSIRO (2021). Australian Water Availability Project (AWAP) Rainfall Grid Data aggregated to month 1900-2020. Data retrieved from Centre for Air pollution, energy and health Research https://cloud.car-dat.org/index.php/apps/files/?dir=/Environment_General/AWAP_GRIDS/AWAP_GRIDS_monthly_rainfall_1900_2020
Climate	clim_relhum_annual	Climate_and_weather_BOM	average_3pm_relative_humidity_1976_2005	Australian Bureau of Meteorology, 2016. Average 9am and 3pm relative humidity Australia 1976-2005. Available from http://www.bom.gov.au/jsp/ncc/climate_averages/relative-humidity/index.jsp [accessed 2020] Downloaded from the Centre for Air pollution, energy and health Research https://cloud.car-

Predictor type	Variable_name	Project ID	Dataset ID	Citation
				dat.org/index.php/apps/files/?dir=/Environment_General/Climate_and_weather_BOM/average_3pm_relative_humidity_1976_2005
Climate	clim_rain_annual	Climate_and_weather_BOM	annual_mean_rainfall_1981_2010	Australian Bureau of Meteorology, 2020. Average annual rainfall across Australia.1960-1991. Available from http://www.bom.gov.au/jsp/ncc/climate_averages/rainfall/index.jsp [accessed 2020] Downloaded from the Centre for Air pollution, energy and health Research https://cloud.car-dat.org/index.php/apps/files/?dir=/Environment_General/Climate_and_weather_BOM/annual_mean_rainfall_1981_2010
Climate	clim_avtmp_annual	Climate_and_weather_BOM	annual_mean_daily_average_temperature_1960_1991	Australian Bureau of Meteorology, 2016. Average annual daily average temperatures across Australia.1960-1991. Available from http://www.bom.gov.au/jsp/ncc/climate_averages/temperature/index.jsp [accessed 2020] Downloaded from the Centre for Air pollution, energy and health Research https://cloud.car-dat.org/index.php/apps/files/?dir=/Environment_General/Climate_and_weather_BOM/annual_mean_daily_average_temperature_1960_1991
Climate	clim_wind_annual	Climate_and_weather_BOM	gridded_annual_wind_velocity_2004_2008	Australian Bureau of Meteorology, 2011. Average annual wind velocity distribution across Australia.2004-2008. Available from http://www.bom.gov.au/jsp/ncc/climate_averages/wind-velocity/index.jsp [accessed 2020] Downloaded from the Centre for Air pollution, energy and health Research https://cloud.car-dat.org/index.php/apps/files/?dir=/Environment_General/Climate_and_weather_BOM/gridded_annual_wind_velocity_2004_2008
Climate	clim_solar_winter	UVR_Solar_BOM	seasonal_daily_avg_solar_uv_aust_1990_2011	Australian Bureau of Meteorology, 2019. Average daily solar ultraviolet (UV) Index for summer (Dec-Feb) and winter (Jun-Aug) averaged over the years 1990-2011. Available from http://www.bom.gov.au/jsp/ncc/climate_averages/solar-exposure/index.jsp [Accessed 2014]. Data downloaded from Centre for Air pollution, energy and health Research. https://cloud.car-dat.org/index.php/apps/files/?dir=/Environment_General/UVR_Solar_BOM/seasonal_daily_avg_solar_uv_aust_1990_2011

Predictor type	Variable_name	Project ID	Dataset ID	Citation
Climate	clim_solar_summer	UVR_Solar_BOM	seasonal_daily_avg_solar_uv_aust_1990_2011	Australian Bureau of Meteorology, 2019. Average daily solar ultraviolet (UV) Index for summer (Dec-Feb) and winter (Jun-Aug) averaged over the years 1990-2011. Available from http://www.bom.gov.au/jsp/ncc/climate_averages/solar-exposure/index.jsp [Accessed 2014]. Data downloaded from Centre for Air pollution, energy and health Research. https://cloud.car-dat.org/index.php/apps/files/?dir=/Environment_General/UVR_Solar_BOM/seasonal_daily_avg_solar_uv_aust_1990_2011
Climate	clim_htng_dg_days_annual	Climate_and_weather_BOM	annual_total_heating_degree_days_1961_1990	Australian Bureau of Meteorology, 2011. Mean monthly and mean annual heating & cooling degree days data (base climatological data sets) 1961-1990. Available from http://www.bom.gov.au/jsp/ncc/climate_averages/degree-days/index.jsp [accessed 2020] Downloaded from the Centre for Air pollution, energy and health Research https://cloud.car-dat.org/index.php/apps/files/?dir=/Environment_General/Climate_and_weather_BOM/annual_total_heating_degree_days_1961_1990

GIS variable definitions

Table S3: Definitions of predictor variables

Predictor type	Dataset ID	variable_name	Unit	Buffers	Variable_definition
Response variable	CARs_National_Air_Pollution_Database	no2_annual	ppb	NA	NA
Response variable	CARs_National_Air_Pollution_Database	pm25_annual	µg/m^3	NA	NA
Response variable	CARs_National_Air_Pollution_Database	pm10_annual	µg/m^3	NA	NA
Satellite-based air pollution estimates	Sat_OMI_NO2_LK_2005_2019	sat_no2	molecules x 10^{15} / cm^2	NA	Annual Column (COL) NO2 satellite data, resolution of 13 x 24 km (nadir). This variable is the same for each month in the year.
Satellite-based air pollution estimates	annual_surface_pm25_concentrations_2005_2018	sat_pm25	µg/m^3	NA	Global satellite-derived surface estimates
Satellite-based air pollution estimates	O3_Multi_Sensor_Reanalysis_MSR2_1979_2019	sat_o3	dobson	NA	Abundance measure
Temporal	AP_monitor_locations	year	NA	NA	Year
Position and Elevation	AP_monitor_locations	lon_metres	m	NA	Hand geocoded government monitoring stations with PM25/NO2/PM10 data. Albers EPSG 3577.
Position and Elevation	AP_monitor_locations	lat_metres	m	NA	Hand geocoded government monitoring stations with PM25/NO2/PM10 data. Albers EPSG 3577.
Position and Elevation	AP_monitor_locations	state	NA	NA	Australian state or territory jurisdiction
Position and Elevation	SRTM_1sec_Smooth_DEM_National	pos_elevation	m	NA	Elevation from Digital Elevation Model SRTM-derived

Predictor type	Dataset ID	variable_name	Unit	Buffers	Variable_definition
Position and Elevation	Australian_maritime_boundaries_2014	pos_distocean	km	NA	Distance to open ocean
Monitor properties	AP_monitor_locations	monitor_type	NA	NA	Field monitor or government regulatory monitor
Monitor properties	AP_monitor_locations	bam_teom	NA	NA	PM measured with BAM method or TEOM method
Land use	apmma_abs_mb	lu_com_{buffer}	%	50m, 100m, 200m, 300m, 400m, 500m, 700m, 1000m, 1500m, 2000m, 3000m, 5000m, 10000m	% coverage in buffer where ABS 2016 MB category was Commercial or Education or Medical/Hospital
Land use	apmma_abs_mb	lu_res_{buffer}	%	50m, 100m, 200m, 300m, 400m, 500m, 700m, 1000m, 1500m, 2000m, 3000m, 5000m, 10000m	% coverage in buffer where ABS 2016 MB category was Residential
Land use	apmma_abs_mb	lu_open_{buffer}	%	50m, 100m, 200m, 300m, 400m, 500m, 700m, 1000m, 1500m, 2000m, 3000m, 5000m, 10000m	% coverage in buffer where ABS 2016 MB category was Water, Primary Production or Other
Land use	apmma_abs_mb	lu_ind_{buffer}	%	50m, 100m, 200m, 300m, 400m, 500m, 700m, 1000m, 1500m, 2000m, 3000m, 5000m, 10000m	% coverage in buffer where ABS 2016 MB category was industrial.
Land use	Geoscape Residential land	lu_bldg_hgt_avg_100	m	100m	Using all buildings in the buffer calculate the mean height in metres. Buildings with a NULL height were assigned the value 0.5m.
Land use	Geoscape Residential land	lu_bldg_foot_area_100	m^2	100m	The total footprint of all buildings that touch the buffer.
Land cover	tree_and_water_coverage_2017_2018	lc_tree_cover_{buffer}	%	50m, 100m, 200m, 300m, 400m, 500m, 700m, 1000m, 1500m, 2000m, 3000m, 5000m, 10000m	Average % of tree cover coverage of raster values in buffer
Land cover	apmma_abs_mb	lc_park_{buffer}	%	50m, 100m, 200m, 300m, 400m, 500m, 700m, 1000m, 1500m, 2000m, 3000m, 5000m, 10000m	% coverage in buffer where ABS 2016 MB category was Parkland
Land cover	tree_and_water_coverage_2017_2018	lc_water_modis_{buffer}	%	50m, 100m, 200m, 300m, 400m, 500m, 700m, 1000m, 1500m, 2000m, 3000m, 5000m, 10000m	% coverage in buffer where MODIS data shows water
Land cover	Landsat_GMIS_v1_Australia_2010	lc_impsa_{buffer}	%	50m, 100m, 200m, 300m, 400m, 500m, 700m, 1000m, 1500m, 2000m, 3000m, 5000m, 10000m	% impervious surface in buffer
Emission sources	Traffic_Load_Australia_2018	em_minrd_invdist	m^{-1}	NA	Inverse distance to nearest minor road

Predictor type	Dataset ID	variable_name	Unit	Buffers	Variable_definition
Emission sources	Traffic_Load_Australia_2018	em_majrd_invdist	m^{-1}	NA	Inverse distance to nearest major road
Emission sources	Traffic_Load_Australia_2018	em_majrd_length_{buffer}	km	50m, 100m, 200m, 300m, 400m, 500m, 700m, 1000m, 1500m, 2000m, 3000m, 5000m, 10000m	Road length in buffer (major road), with dual carriageways counting double length.
Emission sources	Traffic_Load_Australia_2018	em_minrd_length_{buffer}	km	50m, 100m, 200m, 300m, 400m, 500m, 700m, 1000m, 1500m, 2000m, 3000m, 5000m, 10000m	Road length in buffer (minor road)
Emission sources	Traffic_Load_Australia_2018	em_rds_dist	m	NA	Distance to nearest major road
Emission sources	Traffic_Load_Australia_2018	em_rds_int_imputed	vehicles per day (vpd)	NA	Imputed traffic count on nearest major road
Emission sources	Traffic_Load_Australia_2018	em_rds_intinvdist_imputed	vpd/m	NA	Product of traffic count on nearest major road and the inverse distance to nearest major road.
Emission sources	Traffic_Load_Australia_2018	em_all_rds_intinvdist_imputed	vpd/m	NA	Product of traffic count on nearest major or minor road and the inverse distance to that road.
Emission sources	Traffic_Load_Australia_2018	em_all_rds_int_imputed	vpd	NA	Imputed traffic count on the nearest road (major or minor)
Emission sources	household_woodfire_heaters_Australia_2012	em_wood_heat_pct	%	NA	Percentage of households using woodfire as main heating source
Emission sources	NASA_MODIS_burnt_area_2005_2019	em_burned_area_pct_{buffer}	%	10000m, 25000m, 50000m, 100000m, 250000m, 500000m	Percentage of buffer area burnt
Emission sources	NASA_MODIS_active_fires_2005_2015 NASA_MODIS_active_fires_2016_2019	em_fire_dens_fireskm2_{buffer}	$fires/km^2$	10000m, 25000m, 50000m, 100000m 250000m 500000m	Fire density within buffer
Emission sources	NPI_NOX_Australia_2017_2018 NPI_PM25_Australia_2010_2011	em_npi_{pollutant}_dens_siteskm2{buffer}	$sites/km^2$	50m, 100m, 200m, 300m, 400m, 500m, 700m, 1000m, 1500m, 2000m, 3000m, 5000m, 10000m	Number of emission sites per square km for pollutants $PM_{2.5}$, NO_x

Predictor type	Dataset ID	variable_name	Unit	Buffers	Variable_definition
Emission sources	NPI_NOX_Australia_2017_2018<br>NPI_PM25_Australia_2010_2011	em_npi_{pollutant}_dens_kgkm2{buffer}	kg/km^2	50m, 100m, 200m, 300m, 400m, 500m, 700m, 1000m, 1500m, 2000m, 3000m, 5000m, 10000m	Total emission mass (kg) per square km for pollutants $PM_{2.5}$, NO_x
Population	ABS1x1km_Aus_Pop_Grid_2006_2020	pop_dens_{buffer}	persons/km^2	700m, 1000m, 1500m, 2000m, 3000m, 5000m, 10000m	Population density (per square km) in buffer using ABS's estimated 1x1km population grids.
Weather	AWAP_GRIDS_monthly_temperature_1986_2019	wthr_maxtmp_yrly_avg	°C	NA	Annual average of daily maximum temperature
Weather	AWAP_GRIDS_monthly_temperature_1986_2019	wthr_meantmp_yrly_avg	°C	NA	Annual average of daily mean temperature (mean of minimum and maximum temperatures)
Weather	Drought_and_APMMA_locations_2005_2019	wthr_drt_count_months	months	NA	Number of drought months within the calendar year
Weather	Drought_and_APMMA_locations_2005_2019	wthr_drt_max_length	months	NA	Longest drought duration that overlapped that calendar year, only counting months up to the December of that year (i.e. do not count drought months in following years)
Weather	AWAP_GRIDS_monthly_rainfall_1900_2020	wthr_rain_yrly_total	mm	NA	Annual total rainfall
Climate	average_3pm_relative_humidity_1976_2005	clim_relhum_annual	%	NA	Average daily relative humidity at 15:00, averaged yearly, then averaged from 1976-2005
Climate	annual_mean_rainfall_1981_2010	clim_rain_annual	mm	NA	Average of total annual rainfalls, 1960-1991
Climate	annual_mean_daily_average_temperature_1960_1991	clim_avtmp_annual	°C	NA	Average of annual mean daily average temperature, 1960-1991. Daily averages taken from the mean of the min and max daily temps, and averaged over the year.
Climate	gridded_annual_wind_velocity_2004_2008	clim_wind_annual	km/h	NA	Average of annual average wind velocity
Climate	seasonal_daily_avg_solar_uv_aust_1990_2011	clim_solar_winter	MJ/m^2	NA	Seasonal (winter, June to August) mean daily solar exposure 1990-2011

Predictor type	Dataset ID	variable_name	Unit	Buffers	Variable_definition
Climate	seasonal_daily_avg_solar_uv_aust_1990_2011	clim_solar_summer	MJ/m^2	NA	Seasonal (summer, December to February) mean daily solar exposure 1990-2011
Climate	annual_total_heating_degree_days_1961_1990	clim_htng_dg_days_annual	heating degree days	NA	Average of annual total heating degree days (18°C reference temperature), 1961-1990

Descriptive statistics for pollutants monitored by government & project monitors*

Table S4 Descriptive statistics for annual average $PM_{2.5}$ ($\mu g/m^3$)

Year	Number of monitors	Minimum	1st Quartile	Median	Mean	3rd Quartile	Maximum
2005	12	4.7	6.2	6.9	6.8	7.6	8.4
2006	14	5.7	6.4	6.6	6.9	7.1	8.9
2007	12	5.0	5.7	6.1	6.2	6.5	7.9
2008	12	4.4	5.5	5.9	5.9	6.1	7.7
2009	15	5.6	6.2	7.1	7.5	8.3	10.6
2010	16	3.8	4.6	5.7	5.7	6.4	8.3
2011	20	4.5	4.8	5.4	5.9	6.6	9.1
2012	19	4.3	4.8	6.7	6.5	7.8	10.1
2013	20	4.7	6.7	7.9	7.5	8.2	9.4
2014	28	4.2	5.9	7.4	7.2	8.2	13.1
2015	37	3.5	6.6	7.3	7.0	7.7	9.5
2016	48	3.8	6.4	7.3	6.9	7.8	9.7
2017	53	3.9	6.4	7.2	7.0	7.7	9.8
2018	106	3.9	6.3	7.2	7.0	8.1	11.3
All years	117	3.5	5.9	7.0	6.9	7.8	13.1

*2018 included data from project monitors and government monitors.

Table S5 Descriptive statistics for annual average PM_{10} ($\mu g/m^3$)

Year	Number of monitors	Minimum	1st Quartile	Median	Mean	3rd Quartile	Maximum
2005	28	13.5	17.7	19.2	18.9	20.1	24.5
2006	39	13.9	16.1	19.5	19.1	21.3	29.1
2007	45	12.7	15.9	17.4	18.1	20.1	25.9
2008	47	12.4	15.4	17.4	17.3	18.6	24.7
2009	45	17.1	20.2	22.3	22.8	25.1	31.7
2010	46	9.3	14.0	15.6	15.3	17.1	19.1
2011	49	8.7	13.2	15.1	15.2	16.8	21.8
2012	55	9.4	14.6	16.9	17.2	18.9	26.4
2013	59	9.5	15.4	17.3	17.8	19.2	27.8
2014	60	9.9	15.7	17.1	17.4	18.9	24.7
2015	69	9.8	14.5	15.8	16.6	18.5	36.0
2016	66	9.8	14.8	16.0	16.8	18.7	35.1
2017	63	11.6	15.0	17.2	18.0	20.3	36.3
2018	107	9.9	15.8	18.4	18.9	21.2	38.7
All years	134	8.7	15.1	17.3	17.8	19.8	38.7

*2018 included data from project monitors and government monitors.

Table S6 Descriptive statistics for annual average NO_2 (ppb)

Year	Number of monitors	Minimum	1st Quartile	Median	Mean	3rd Quartile	Maximum
2005	35	2.2	6.6	8.8	8.9	10.8	16.7
2006	78	2.1	8.1	10.8	10.5	12.7	22.5
2007	45	1.9	5.6	7.3	7.9	10.2	16.6
2008	79	1.8	7.3	9.6	9.2	10.7	15.5
2009	42	2.9	5.6	6.9	7.8	9.9	17.6
2010	45	2.1	4.9	6.9	7.3	8.9	15.8
2011	48	1.8	5.2	7.1	7.4	9.2	16.9
2012	43	1.7	5.2	6.8	7.3	8.8	16.1
2013	48	1.8	5.0	6.4	7.1	8.8	16.6
2014	98	1.6	5.8	7.9	8.0	9.8	17.7
2015	52	1.5	4.7	6.9	7.1	8.8	16.5
2016	50	1.5	4.4	6.5	6.8	8.7	14.3
2017	47	1.4	4.9	6.4	7.2	9.8	15.3
2018	92	1.5	4.3	6.2	6.5	8.4	15.8
All years	198	1.4	5.2	7.6	7.9	9.9	22.5

*2006, 2008, 2014 and 2018 included data from project monitors and government monitors.

Spatial Cross validation (CV) results for base learner & meta-learner evaluations

Table S7 Spatial cross-validation (CV) results for base learner and meta-learner evaluations for $PM_{2.5}$, PM_{10}, and NO_2

a) $PM_{2.5}$			
		Training (N=372) 10-fold spatial CV	
**Base learner models**	**Variables**	**R^2**	**RMSE ($\mu g/m^3$)**
GBM	79	0.32	1.18
XGBoost	79	0.27	1.23
Random Forest	79	0.38	1.16
**Meta-learner models (2nd stage)**	**Weights for NNLS (ensemble)**	**R^2**	**RMSE ($\mu g/m^3$)**
GLMNET	0.975	0.43	1.07
Random Forest	0	0.40	1.1
XGBoost	0.002	0.36	1.19
GBM	0.023	0.39	1.11
**b) PM_{10}**			
		Training (N=702) 10-fold spatial CV	
**Base-learner models**	**Variables**	**R^2**	**RMSE ($\mu g/m^3$)**
GBM	79	0.60	2.46
XGBoost	79	0.59	2.49
Random Forest	79	0.57	2.76
**Meta-learner models (2nd stage)**	**Weights for NNLS (ensemble)**	**R^2**	**RMSE ($\mu g/m^3$)**
GLMNET	0.932	0.62	2.38
Random Forest	0.069	0.57	2.53
XGBoost	0	0.51	2.78
GBM	0	0.59	2.46
**c) NO_2**			
		Training (N=722) 10-fold spatial CV	
**Base-learner models**	**Variables**	**R^2**	**RMSE (ppb)**
GBM	79	0.74	1.68
XGBoost	79	0.72	1.75
Random Forest	79	0.71	1.83
**Meta-learner models (2nd stage)**	**Weights for NNLS (ensemble)**	**R^2**	**RMSE (ppb)**
GLMNET	0.726	0.81	1.46
Random Forest	0.274	0.78	1.56
XGBoost	0	0.74	1.71
GBM	0	0.79	1.52

Table S7 presents the base and meta learner results using 10-fold spatial CV (leave-locations-out). We ran the spatial CV for the base and meta learner models, using the same hyperparameters of the best performing model as selected by random 10-fold CV. These results indicate model performance under the spatial CV scheme at the stages where cross-validation is actually applied. The ensemble weighting is deterministic and not subject to the same 10-fold splitting as the base and meta models, and so those metrics for the DEML are not shown as they would conflate two different validation frameworks.

Relative influence of predictors for best performing base-learner models for prediction of $PM_{2.5}$, PM_{10} and NO_2 (refer to Table S2 and S3 for variable definitions)

Figure S1-Relative influence of XGBoost base-learner (best performing base learner ML model) for $PM_{2.5}$ prediction

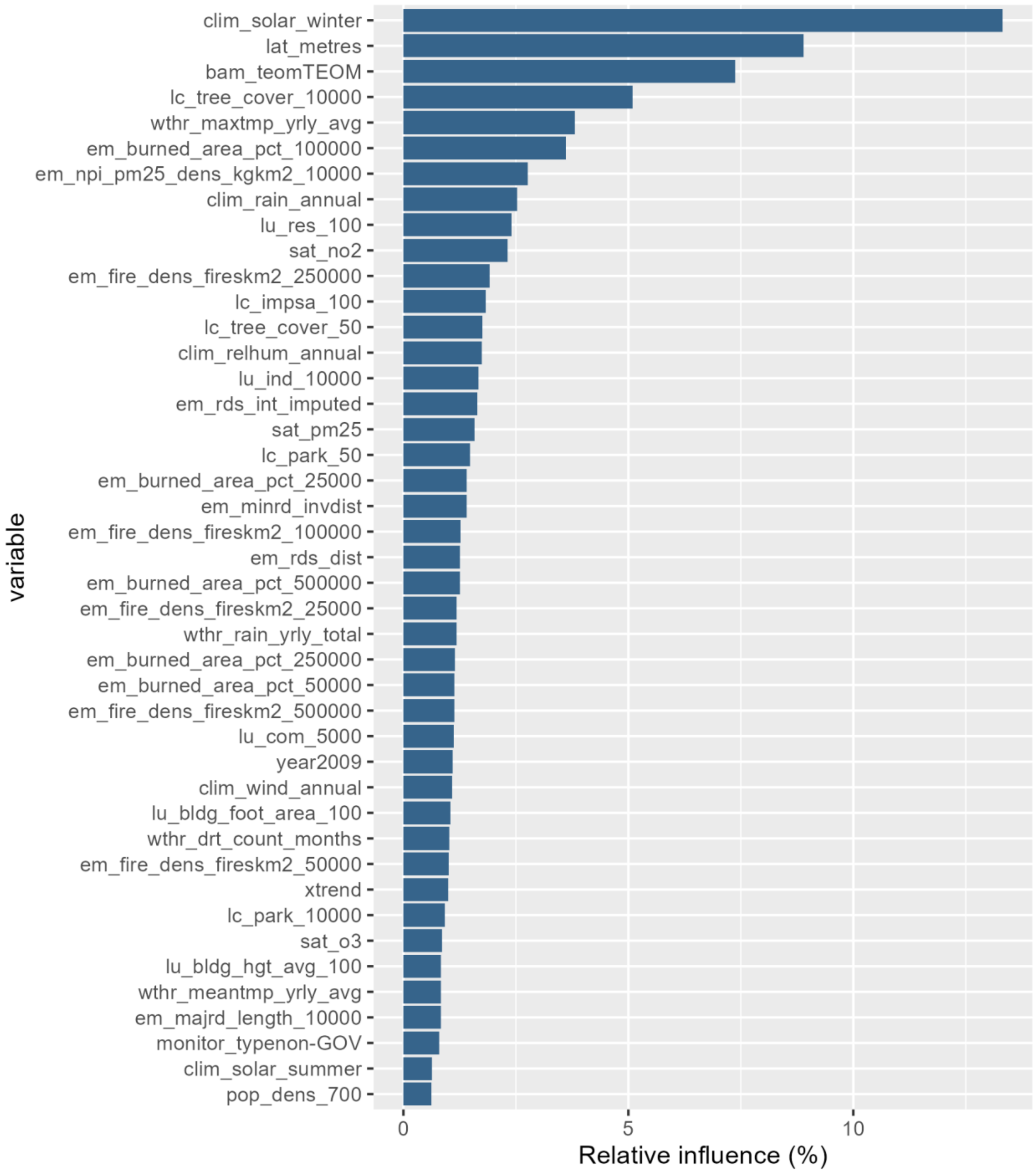

Figure S2-Relative influence of GBM base-learner (best performing base learner ML model) for PM_{10} prediction

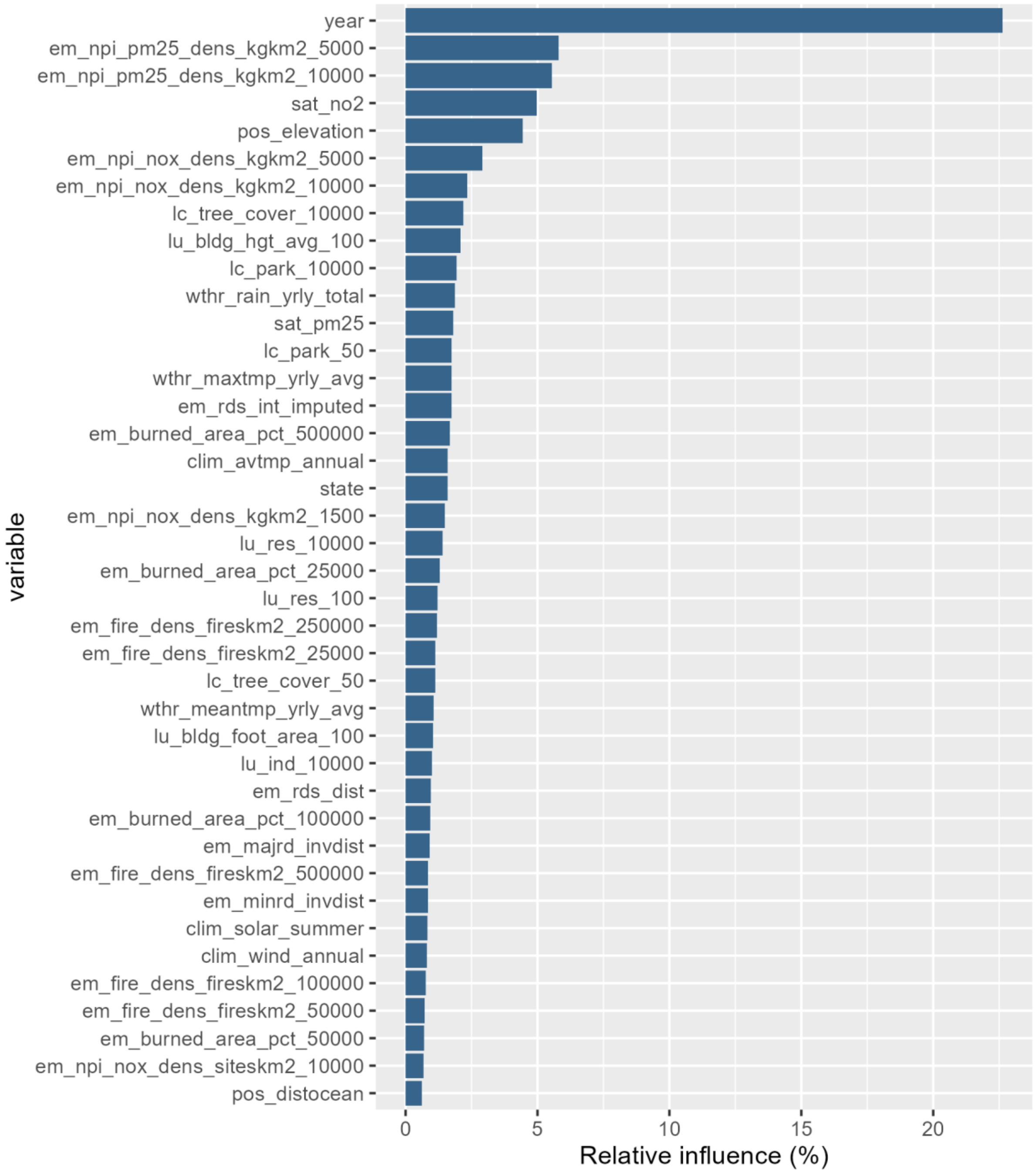

Figure S3-Relative influence of GBM base-learner (best performing base learner ML model) for NO_2 prediction

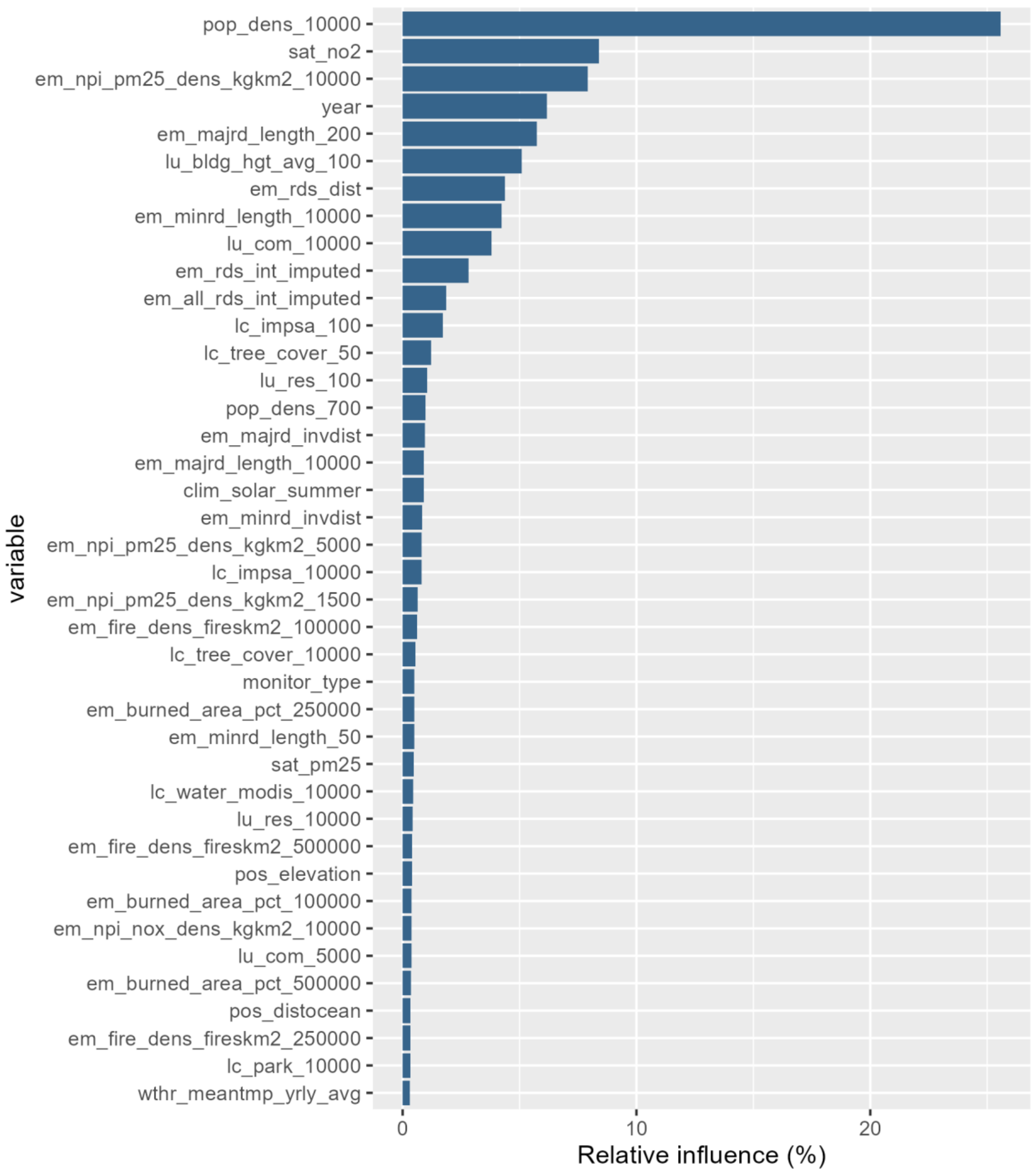

Supplement: Supplementary file 1 [file es6c02076_si_001.pdf]
